# Risk factors for bronchiolitis hospitalization in infants: A French nationwide retrospective cohort study over four consecutive seasons (2009-2013)

**DOI:** 10.1371/journal.pone.0229766

**Published:** 2020-03-06

**Authors:** Brigitte Fauroux, Jean-Michel Hascoët, Pierre-Henri Jarreau, Jean-François Magny, Jean-Christophe Rozé, Elie Saliba, Michaël Schwarzinger

**Affiliations:** 1 Pediatric Noninvasive Ventilation and Sleep Unit, Necker-Enfants malades Hospital, Paris, France; 2 Université de Paris, VIFASOM, Paris, France; 3 Regional Maternity CHRU, University of Lorraine, Nancy, France; 4 Université de Paris, Paris, France; 5 Department of Neonatal Medicine and Intensive Care Unit of Port-Royal, Cochin Hospital, AP-HP, Paris, France; 6 Department of Neonatology, Necker-Enfants malades University Hospital, Paris, France; 7 Department of Neonatology, University Hospital, Nantes, France; 8 Department of Neonatology, Clocheville University Hospital, Tours, France; 9 Translational Health Economics Network (THEN), Paris, France; 10 Infection Antimicrobials Modeling & Evolution (IAME), UMR 1137, Institut National de la Santé et de la Recherche Médicale (INSERM), Paris, France; 11 Université Paris Diderot, Sorbonne Paris Cité, Paris, France; Ben-Gurion University of the Negev, UNITED STATES

## Abstract

**Objectives:**

Large studies are needed to update risk factors of bronchiolitis hospitalization. We performed a nationwide analysis of hospitalization rates for bronchiolitis over four consecutive bronchiolitis seasons to identify underlying medical disorders at risk of bronchiolitis hospitalization and assess their frequency.

**Methods:**

Data were retrieved from the French National Hospital Discharge database. Of all infants discharged alive from maternity wards from January 2008 to December 2013 in France (N = 3,884,791), we identified four consecutive cohorts at risk of bronchiolitis during the seasons of 2009–2010 to 2012–2013. The main outcome was bronchiolitis hospitalization during a season. Individual risk factors were collected.

**Results:**

Among infants, 6.0% were preterm and 2.0% had ≥1 chronic condition including 0.2% bronchopulmonary dysplasia (BPD) and 0.2% hemodynamically significant congenital heart disease (HS-CHD). Bronchiolitis hospitalization rates varied between seasons (min: 1.26% in 2010–2011; max: 1.48% in 2012–2013; p<0.001). Except omphalocele, the following conditions were associated with an increased risk for bronchiolitis hospitalization: solid organ (9.052; 95% CI, 4.664–17.567) and stem cell transplants (6.012; 95% CI, 3.441–10.503), muscular dystrophy (4.002; 95% CI, 3.1095–5.152), cardiomyopathy (3.407; 95% CI, 2.613–4.442), HS-CHD (3.404; 95% CI, 3.153–3.675), congenital lung disease and/or bronchial abnormalities, Down syndrome, congenital tracheoesophageal fistula, diaphragmatic hernia, pulmonary hypertension, chromosomal abnormalities other than Down syndrome, hemodynamically non-significant CHD, congenital abnormalities of nervous system, cystic fibrosis, cleft palate, cardiovascular disease occurring during perinatal period, and BPD.

**Conclusion:**

Besides prematurity, BPD, and HS–CHD, eighteen underlying conditions were associated with a significant increased risk for bronchiolitis hospitalization in a nationwide population.

## Introduction

Bronchiolitis is the most common seasonal viral respiratory disorder and the leading cause of hospital admission due to viral infection in the first 12 months of life [1–4]. Respiratory syncytial virus (RSV) represents the most common cause of bronchiolitis, followed by other viruses, such as rhinovirus, influenza, or metapneumovirus [1,3,4]. It is well-established that premature infants and those with bronchopulmonary dysplasia (BPD) or congenital heart disease (CHD) are at high-risk for severe bronchiolitis requiring hospital admission during epidemic season [5–7]. Recently, D. Verhoeven notes that although some of the morbidity associated with RSV in neonates is due to immunological maturation, the rapid development of the immune system right after birth suggests that each age group of infants may respond to the virus in different ways [8]. Infants with chronic conditions, such as Down syndrome, congenital malformations, especially pulmonary and airway anomalies, cystic fibrosis, and neuromuscular diseases, may also be associated with an increased vulnerability to severe bronchiolitis [9]. However, a recent systematic review has highlighted the need for large studies to better identify, update, and estimate risk factors associated with hospital admission for bronchiolitis, especially those including infants with rare underlying medical disorders in a real-life setting [5–7,9]. In the area of innovative therapies for RSV infection bronchiolitis, it is also crucial to have solid data on risk factors and susceptible populations for an optimal use of new resources.

The aims of the present study were to 1) perform a nationwide analysis of the hospitalization rate for bronchiolitis in infants over four consecutive seasons using nationwide hospital discharge data, and 2) identify independent risk factors for bronchiolitis hospitalization in this large cohort.

## Methods

The present study was a retrospective cohort study over four consecutive bronchiolitis seasons.

### Data source

Data were retrieved from the French National Hospital Discharge database (“Programme de Médicalisation des Systèmes d’Information”; PMSI). This database provides exhaustive medico-administrative information about all patients admitted to public and private hospitals in France. Hospital births represent more than 95% of all births in France [10]. For each hospital birth, a standard discharge abstract is recorded and encompasses the following characteristics: birth weight, gestational age (GA) in weeks, primary and associated discharge diagnoses encoded using the 10^th^ revised edition of the *International Classification of Diseases* (ICD–10), medical procedures, admission to the neonatal intensive care unit (NICU), length of stay and in-hospital death. Using an anonymous unique identifier, any rehospitalization following maternity discharge can be followed over time [2,11].

### Study population

All infants discharged alive from maternity wards from January 1, 2008, to December 31, 2013, in metropolitan France were initially included in the study. Infants discharged with missing birth weight or discharged after March 31, 2013, were excluded.

**Fig 1:**
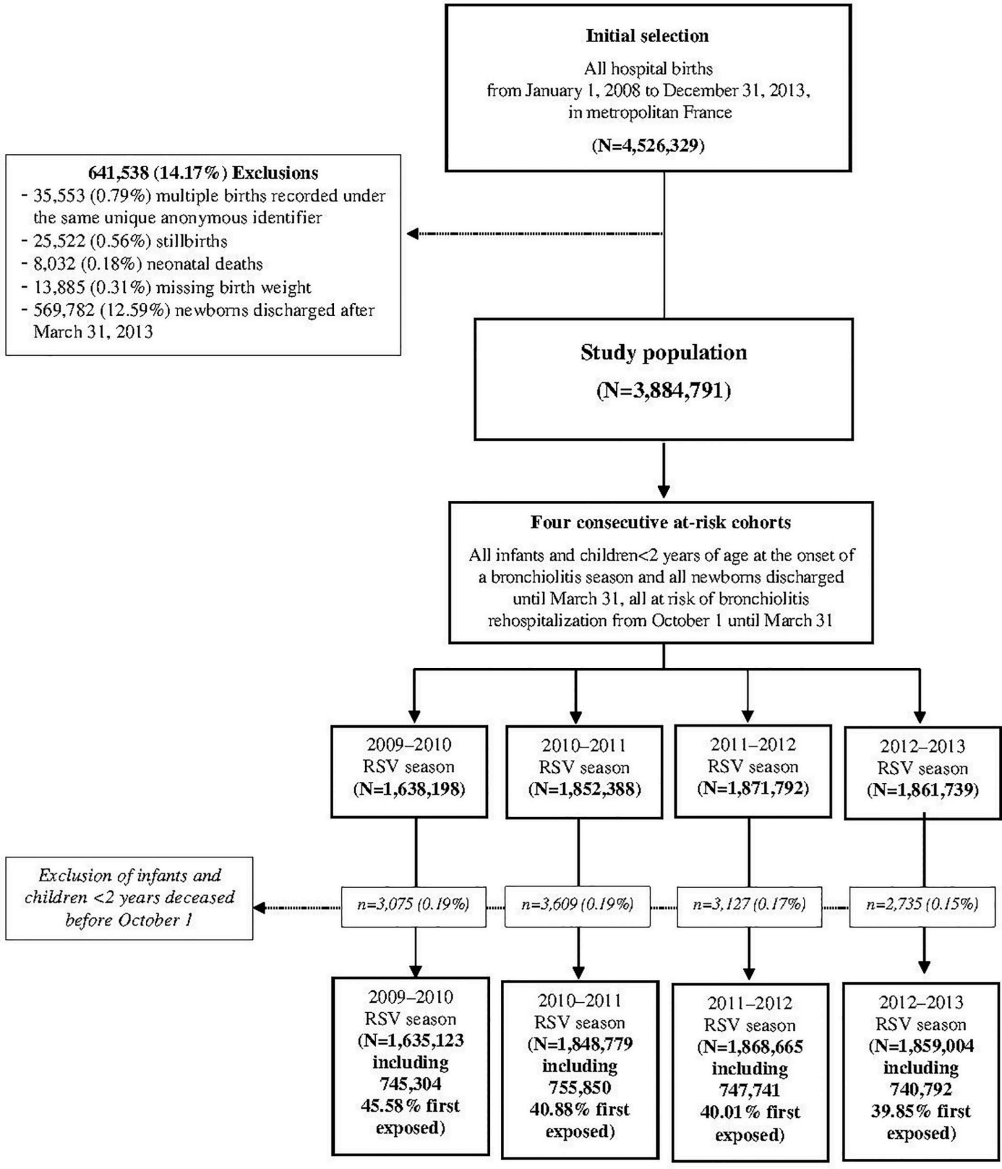
Flow chart of sample selection. Abbreviations: RSV, respiratory syncytial virus.

The study population was structured into four consecutive cohorts at risk of bronchiolitis during the seasons of 2009–2010 to 2012–2013 (Fig 1). The bronchiolitis season usually lasts from early October to late March of the following year in France [3]. Accordingly, each cohort was composed of all infants <2 years of age on October 1, as well as all newborns discharged until March 31. In theory, all infants could be consecutively exposed to 2 or 3 seasons depending on their birthdate in April to September or October to March, respectively. Because hospital discharge data were left-truncated on January 1, 2008 and right-truncated on March 31, 2013, infants could be followed at different ages in 1, 2, or 3 consecutive cohorts. In particular, infants older than 12 months at October 1, 2009, and infants born after March 31, 2012, were followed over a single season, while infants born during the season of 2009–2010 or 2010–2011 were followed at different ages in three consecutive cohorts.

### Outcomes

The main outcome was hospitalization for acute bronchiolitis (ICD-10: J21) during a bronchiolitis season. The following individual risk factors were collected: single or multiple births, birth weight, GA, age at season onset, underlying medical disorders, and history of bronchiolitis and/or low respiratory tract infection (LRTI). Unlike birth weight, GA was not mandatory in standard discharge abstracts until March 1, 2009, and some maternity wards did not record GA. Multivariate regression was therefore used to impute missing completely at random data on GA (11.9%) based on gender, birth weight, and main characteristics of neonatal stay (i.e., length of hospital stay, admission to NICU, and in-hospital death) [12,13]. GA was categorized into extremely preterm (22–27 GA), very preterm (28–32 GA), moderate preterm (33–36 GA), full-term (37–41 GA), and post-term (≥42 GA) [14]. The fifth and tenth percentiles of birth weight were calculated by gender and GA for the study population (S1 Table**)** and birth weight was categorized into intrauterine growth restriction and other very small for GA (birth weight <5%) or small for GA (birth weight: 5–10%).

Age at season onset was calculated by the time from neonatal discharge to October 1 and infants and children were grouped into seven age categories: >18 months; 13–18 months; 7–12 months; 4–6 months (April–June); 1–3 months (July–September); born at onset of the bronchiolitis season (October–December); and born at the end of the bronchiolitis season (January–March). Infants discharged from the NICU at onset of the bronchiolitis season were also identified.

Nineteen chronic conditions that have been associated with an increased risk for bronchiolitis were retrieved using a medical literature search (S1 Appendix) [9,15,16]. All the corresponding ICD–10 codes were collected from hospital records, including BPD and CHD, according to the criteria from the Anatomic and Clinical Classification of Congenital Heart Disease and corresponding ICD–10 codes [17]. Hemodynamically significant CHD (HS–CHD) was identified by any cardiac surgery. Preterm infants with patent ductus arteriosus requiring a treatment were reported as HS–CHD. A history of bronchiolitis (and otherwise LRTI) was identified by any record of bronchiolitis (and otherwise LRTI) before the bronchiolitis season (i.e., during the neonatal hospital stay or before October 1). In addition, the following maternal characteristics were identified from the neonatal hospital stay: “smoking during pregnancy”, “cardiovascular and respiratory diseases”, and “diabetes”.

All ICD–10 codes are detailed in S2 Appendix.

### Confounding factors

Postal codes of maternity wards were gathered into five main French regions to adjust for geographical area. Furthermore, environmental risk factors comprise smoking habits and socioeconomic status of parents, which are not described in the standard discharge abstracts of infants. We calculated 7 population statistics related to these environmental risk factors by postal code of residency (n = 5,645): mean age at first pregnancy; hospitalization rates for in vitro fertilization; hospitalization rates for tobacco addiction, chronic obstructive pulmonary disease, or asthma; percentage of women with at least a high school diploma; and living in rural areas. Population statistics were linked to the postal code of maternal residency and used as proxies to control for environmental risk factors of the infant.

### Statistical analysis

Hospitalization rates for bronchiolitis were calculated by bronchiolitis season with use of the number of infants admitted to hospital for bronchiolitis during the season (numerator) among all infants at risk on October 1 of each season (denominator). Multivariate logistic regression was applied to identify risk factors for bronchiolitis hospitalization. Generalized Estimating Equations (GEE) with exchangeable correlation structure was used to take into account repeated measurements at different ages of the infant. Associations between the risk of bronchiolitis hospitalization, seasons, and individual risk factors were expressed by adjusted odds ratios (ORs) and 95% confidence intervals (CIs).

Sensitivity analyses were performed on the case definition of bronchiolitis using hospitalization for severe bronchiolitis (i.e., bronchiolitis requiring a NICU admission, respiratory support or associated with respiratory insufficiency), RSV infection (i.e., LRTI related to RSV), and LRTI during a bronchiolitis season [18–20]. Sensitivity analyses were also performed on the effect of repeated measurements by selecting infants at first bronchiolitis season exposure.

All analyses involved the use of SAS statistical software, version 9·4 (SAS Institute, Cary, NC, USA).

### Ethics and consent

The study was approved by the French National Commission for Data Protection (CNIL DE–2015–025) who granted access to the French National Hospital Discharge database for the years 2008 to 2013. The requirement for informed consent was waived because the study used de–identified data.

## Results

### Characteristics of the study population

The study population comprised 3,884,791 infants (Fig 1) after excluding 13,885 (0.31%) infants with missing birth weight. Boys and girls were almost evenly distributed across bronchiolitis seasons (Table 1).

**Table 1:** Characteristics of infants and children <2 years at-risk over four consecutive bronchiolitis seasons (N = 7,211,571).

Characteristics, n (%)	2009–2010	2010–2011	2011–2012	2012–2013
**Cohort at- risk on October 1, N**^**a**^	1,635,123	1,848,779	1,868,665	1,859,004
**Characteristics of the infant and delivery:**				
**Male sex**	837,913 (51.24)	945,688 (51.15)	954,386 (51.07)	948,580 (51.03)
**Multiple birth**	22,676 (1.39)	25,726 (1.39)	26,517 (1.42)	26,495 (1.43)
**Gestational age:**				
Extremely preterm (22–27 GA)	2,193 (0.13)	2,400 (0.13)	2,485 (0.13)	2,495 (0.13)
Very preterm (28–32 GA)	13,015 (0.80)	14,281 (0.77)	14,639 (0.78)	14,572 (0.78)
Moderate preterm (33–36 GA)	81,220 (4.97)	93,526 (5.06)	95,515 (5.11)	96,174 (5.17)
Full-term (37–41 GA)	1,528,984 (93.51)	1,724, 815 (93.29)	1,740,782 (93.16)	1,729,616 (93.04)
Post-term (≥42 GA)	9,711 (0.59)	13,757 (0.74)	15,244 (0.82)	16,147 (0.87)
**Growth restriction:**				
Intrauterine growth restriction	6,135 (0.38)	7,339 (0.40)	7,352 (0.39)	7,107 (0.38)
Very small for GA (birth weight <5%)	55,060 (3.37)	81,090 (4.39)	88,934 (4.76)	88,980 (4.79)
Small for GA (birth weight: 5–10%)	55,652 (3.40)	81,592 (4.41)	89,506 (4.79)	89,755 (4.83)
**Time elapsed from neonatal discharge to October 1:**				
Second or third exposure to a bronchiolitis season				
19–24 months (19–21 in 2009–2010)	173,282 (10.60)	348,781 (18.87)	366,043 (19.59)	371,491 (19.98)
13–18 months	364,140 (22.27)	374,575 (20.26)	379,513 (20.31)	379,723 (20.43)
7–12 months	352,397 (21.55)	369,573 (19.99)	375,368 (20.09)	366,998 (19.74)
First exposure to a bronchiolitis season				
4–6 months (April–June)	181,082 (11.07)	185,080 (10.01)	184,839 (9.89)	181,969 (9.79)
1–3 months (July–September)	193,695 (11.85)	194,679 (10.53)	195,182 (10.44)	192,119 (10.33)
RSV season onset (October–December)	187,132 (11.44)	195,191 (10.56)	187,144 (10.01)	190,253 (10.23)
RSV season end (January–March)	183,395 (11.22)	180,900 (9.78)	180,576 (9.66)	1,76,451 (9.49)
**Discharge from NICU (October–December)**	8,194 (0.50)	8,727 (0.47)	8,415 (0.45)	8,654 (0.47)
**History of severe respiratory infection:**				
Bronchiolitis	25,091 (1.53)	36,392 (1.97)	39,515 (2.11)	41,234 (2.22)
Other LRTI	6,142 (0.38)	8,592 (0.46)	7,499 (0.40)	7,219 (0.39)
**Underlying medical disorders:**				
Bronchopulmonary dysplasia^b^	3,375 (0.21)	3,619 (0.20)	3,825 (0.20)	3,992 (0.21)
Congenital heart disease^c^				
Hemodynamically significant CHD (surgery)^c^	2,856 (0.17)	3,096 (0.17)	3,103 (0.17)	2,721 (0.15)
Hemodynamically non-significant CHD^c^	10,054 (0.61)	11,140 (0.60)	11,460 (0.61)	11,949 (0.64)
Other underlying medical disorders^b^	16,818 (1.03)	18,918 (1.02)	19,836 (1.06)	20,934 (1.13)
Pulmonary hypertension	363 (0.02)	377 (0.02)	414 (0.02)	412 (0.02)
Congenital lung disease and/or bronchial abnormalities	497 (0.03)	573 (0.03)	592 (0.03)	566 (0.03)
Congenital tracheoesophageal fistula	343 (0.02)	406 (0.02)	408 (0.02)	412 (0.02)
Cystic fibrosis	481 (0.03)	550 (0.03)	551 (0.03)	489 (0.03)
Cardiovascular disease occurring during the perinatal period without CHD identified in the follow-up	10,054 (0.61)	11,542 (0.62)	12,614 (0.68)	14,037 (0.76)
Cardiomyopathy	211 (0.01)	233 (0.01)	242 (0.01)	224 (0.01)
Diaphragmatic hernia	295 (0.02)	302 (0.02)	261 (0.01)	211 (0.01)
Omphalocele	264 (0.02)	258 (0.01)	237 (0.01)	274 (0.01)
Muscular dystrophy	250 (0.02)	246 (0.01)	238 (0.01)	178 (0.01)
Congenital abnormalities of the nervous system	761 (0.05)	822 (0.04)	793 (0.04)	768 (0.04)
Cleft palate	1,925 (0.12)	2,117 (0.11)	2,059 (0.11)	2,027 (0.11)
Down syndrome	876 (0.05)	1,029 (0.06)	1,109 (0.06)	1,120 (0.06)
Other chromosomal abnormality	924 (0.06)	952 (0.05)	840 (0.04)	725 (0.04)
HIV infection	111 (0.01)	116 (0.01)	101 (0.01)	74 (0.00)
Solid organ transplant	21 (0.00)	23 (0.00)	27 (0.00)	26 (0.00)
Stem cell transplant	43 (0.00)	45 (0.00)	44 (0.00)	49 (0.00)
**Maternal disorders during pregnancy:**				
Maternal smoking	31,426 (1.92)	37,752 (2.04)	39,745 (2.13)	42,559 (2.29)
Cardiovascular or respiratory diseases	1,134 (0.07)	1,255 (0.07)	1,269 (0.07)	1,264 (0.07)
Diabetes mellitus	31,647 (1.94)	40,497 (2.19)	45,558 (2.44)	49,635 (2.67)

Abbreviations: CHD, congenital heart disease; GA, gestational age; ICD–10, 10^th^ revised edition of the *International Classification of Diseases*; LRTI, lower respiratory tract infection; NICU, neonatal intensive care unit; RSV, respiratory syncytial virus.

^a^Depending on birthdate, a newborn may be at-risk in one to three consecutive bronchiolitis seasons. For instance, an infant born in March 1, 2010 was at-risk in the 2009–2010 season (age category: RSV season end), 2010–2011 season (age category: 7–12 months), and 2011–2012 season (age category: 19–24 months).

^b^ICD–10 codes were identified from hospital records.

^c^CHD was identified according to the criteria from the Anatomic and Clinical Classification of Congenital Heart Disease and corresponding to validated ICD–10 codes [17]. Cardiac surgery was identified as hemodynamically significant CHD.

Preterm infants accounted for about 6.0% of live-born infants on October 1 each year: 0.1% extremely preterm, 0.8% very preterm, and 5.1% moderate preterm. Infants with ≥1 diagnosis out of nineteen chronic conditions under study represented approximately 2.0% of the study population. Approximately 0.2% of live-born infants on October 1 of each year had BPD and HS–CHD. Hemodynamically non-significant CHD and other underlying medical disorders were present in 0.6% and 1.1% of infants, respectively. A history of bronchiolitis and LRTI, respectively, was noted in 2.0% and 0.4% of the infants on October 1. Maternal smoking during pregnancy was reported for 2.1% of the infants.

### Bronchiolitis hospitalization rates

Hospitalization rates for bronchiolitis varied significantly between seasons, with a higher rate in 2012–2013 (1.48%) compared with 2010–2011 (1.26%; p < .001; Table 2).

**Table 2:** Hospital admissions for bronchiolitis, severe bronchiolitis, RSV infection, and acute LRTI over four consecutive bronchiolitis seasons (N = 7,211,571).

Outcomes, n (%)	2009–2010	2010–2011	2011–2012	2012–2013
**Cohort at-risk on October 1, N**^**a**^	1,635,123	1,848,779	1,868,665	1,859,004
**Bronchiolitis (RSV or not)**	23,466 (1.44)	23,218 (1.26)	25,352 (1.36)	27,458 (1.48)
**Sensitivity analyses:**				
**Severe bronchiolitis (RSV or not):**	5,970 (0.37)	6,331 (0.34)	7,310 (0.39)	8,272 (0.44)
Admission in NICU	1,810 (30.32)	1,955 (30.88)	2,365 (32.35)	2,608 (31.53)
Respiratory support^b^	2,719 (45.54)	2,945 (46.52)	3,333 (45.60)	3,861 (46.68)
Respiratory insufficiency (ICD–10 code)	1,441 (24.14)	1,431 (22.60)	1,612 (22.05)	1,803 (21.80)
**RSV infection**^**c**^	12,195 (0.75)	11,745 (0.64)	12,611 (0.67)	13,964 (0.75)
**Acute LRTI**	29,676 (1.81)	28,706 (1.55)	30,632 (1.64)	32,955 (1.77)

Abbreviations: ICD–10, 10^th^ revised edition of the *International Classification of Diseases*; LRTI, lower respiratory tract infection; NICU, neonatal intensive care unit; RSV, respiratory syncytial virus.

^a^Depending on birthdate, a newborn may be at risk in one to three consecutive bronchiolitis seasons. For instance, an infant born in March 1, 2010 was at risk in the 2009–2010 season (age category: RSV season end), 2010–2011 season (age category: 7–12 months), and 2011–2012 season (age category: 19–24 months).

^b^Procedure was coded using the French Classification of Medical Procedures.

^c^Of 50,515 RSV infections, 48,632 (96·3%) were recorded as bronchiolitis due to RSV

Data are expressed as n (%).

Similar seasonal trends were found when strengthening the definition of bronchiolitis to severe bronchiolitis or RSV infection (mostly (96.3%) recorded as bronchiolitis due to RSV). A seasonal peak was observed in 2009–2010 when broadening the definition of bronchiolitis to all 121,969 admissions for acute LRTI.

### Risk factors for bronchiolitis hospitalization

All potential risk factors were associated with the risk for bronchiolitis hospitalization by season (S2 Table) and integrated along season into the multivariate analysis (Table 3).

**Table 3:** Risk factors of hospital admission for bronchiolitis–multivariate analyses of 99,494 hospital admissions for bronchiolitis (N = 7,211,571).

Variables	OR (95%CI)	P-value
**Bronchiolitis seasons (ref: 2011–2012):**		
2009–2010	0.96 (0.94–0.98)	< .0001
2010–2011	0.90 (0.88–0.92)	< .0001
2012–2013	1.08 (1.06–1.10)	< .0001
**Characteristics of the infant and delivery:**		
**Male sex**	1.27 (1.25–1.29)	< .0001
**Multiple birth**	1.24 (1.18–1.29)	< .0001
**Gestational age (ref: ≥37 GA):**		
Extremely preterm (22–27 GA)	2.60 (2.32–2.92)	< .0001
Very preterm (28–32 GA)	3.11 (2.96–3.27)	< .0001
Moderate preterm (33–36 GA)	1.96 (1.91–2.01)	< .0001
**Growth restriction:**		
Intrauterine growth restriction	1.32 (1.22–1.44)	< .0001
Very small for GA (birth weight <5%)	1.14 (1.11–1.18)	< .0001
Small for GA (birth weight: 5–10%)	1.06 (1.03–1.09)	< .001
**Time elapsed from neonatal discharge to October1 (ref:7–12 months):**		
19–24 months (19–21 in 2009–2010)	0.19 (0.18–0.20)	< .0001
13–18 months	0.42 (0.40–0.43)	< .0001
3–6 months (April–June)	2.27 (2.21–2.34)	< .0001
1–3 months (July–September)	4.35 (4.24–4.46)	< .0001
**RSV season onset (October–December)**	6.59 (6.44–6.75)	< .0001
RSV season end (January–March)	1.43 (1.38–1.47)	< .0001
Discharge from NICU (October–December)	1.16 (1.11–1.22)	< .0001
**History of severe respiratory infection:**		
Bronchiolitis	4.05 (3.88–4.23)	< .0001
Other LRTI	2.57 (2.35–2.82)	< .0001
**Underlying medical disorders:**		
Bronchopulmonary dysplasia^a^	1.35 (1.23–1.48)	< .0001
Congenital heart disease^b^		
Hemodynamically significant CHD (surgery)	3.37 (3.08–3.69)	< .0001
Hemodynamically non-significant CHD	2.10 (1.98–2.22)	< .0001
Other underlying medical disorders^a^		
Pulmonary hypertension	2.55 (2.01–3.24)	< .0001
Congenital lung disease and/or bronchial abnormalities	3.30 (2.68–4.06)	< .0001
Congenital tracheoesophageal fistula	3.00 (2.40–3.74)	< .0001
Cystic fibrosis	1.99 (1.55–2.56)	< .0001
Cardiovascular disease occurring during the perinatal period without CHD identified in the follow-up	1.36 (1.27–1.45)	< .0001
Cardiomyopathy	3.38 (2.47–4.62)	< .0001
Diaphragmatic hernia	3.06 (2.26–4.13)	< .0001
Omphalocele	1.05 (0.68–1.62)	.83
Muscular dystrophy	4.35 (3.15–6.01)	< .0001
Congenital abnormalities of the nervous system	1.59 (1.29–1.97)	< .0001
Cleft palate	1.46 (1.25–1.71)	<·0001
Down syndrome	3.29 (2.88–3.76)	< .0001
Other chromosomal abnormalities	2.29 (1.92–2.73)	< .0001
HIV infection	1.89 (1.09–3.29)	.023
Solid organ transplant	9.76 (4.11–23.15)	< .0001
Stem cell transplant	5.83 (3.00–11.33)	< .0001
**Maternal disorders during pregnancy:**		
Maternal smoking	1.42 (1.36–1.47)	< .0001
Cardiovascular and respiratory diseases	1.27 (1.03–1.57)	.023
Diabetes mellitus	1.10 (1.05–1.14)	< .0001

Abbreviations: CI, confidence interval; CHD, congenital heart disease; ICD–10, 10^th^ revised edition of the *International Classification of Diseases*; LRTI, lower respiratory tract infection; NICU, neonatal intensive care unit; OR, odds ratio; RSV, respiratory syncytial virus.

^a^ICD–10 codes were identified by hospital records.

^b^CHD was identified according to the criteria from the Anatomic and Clinical Classification of Congenital Heart Disease and corresponding to validated ICD–10 codes [17] Cardiac surgery was identified as hemodynamically significant CHD.

Multivariate logistic regression models were developed using Generalized Estimating Equations (GEE) with exchangeable correlation structure. Odds ratios were adjusted for were adjusted for all covariates as well as region of maternity wards and environmental risk factors.

All risk factors except omphalocele were independent risk factors for hospital admission for bronchiolitis. Boys had a 27% increased risk of admission for bronchiolitis compared with girls. Multiple birth, lower GA, a lower birth weight for GA, and birth during or close to the onset of the bronchiolitis season were associated with an increased risk of admission for bronchiolitis. Tobacco exposure during pregnancy, but also maternal cardiovascular and respiratory diseases, and maternal diabetes mellitus were associated with an increased risk of bronchiolitis hospitalization. A history of bronchiolitis and/or LRTI was associated with an increased risk of bronchiolitis hospitalization.

**Fig 2:**
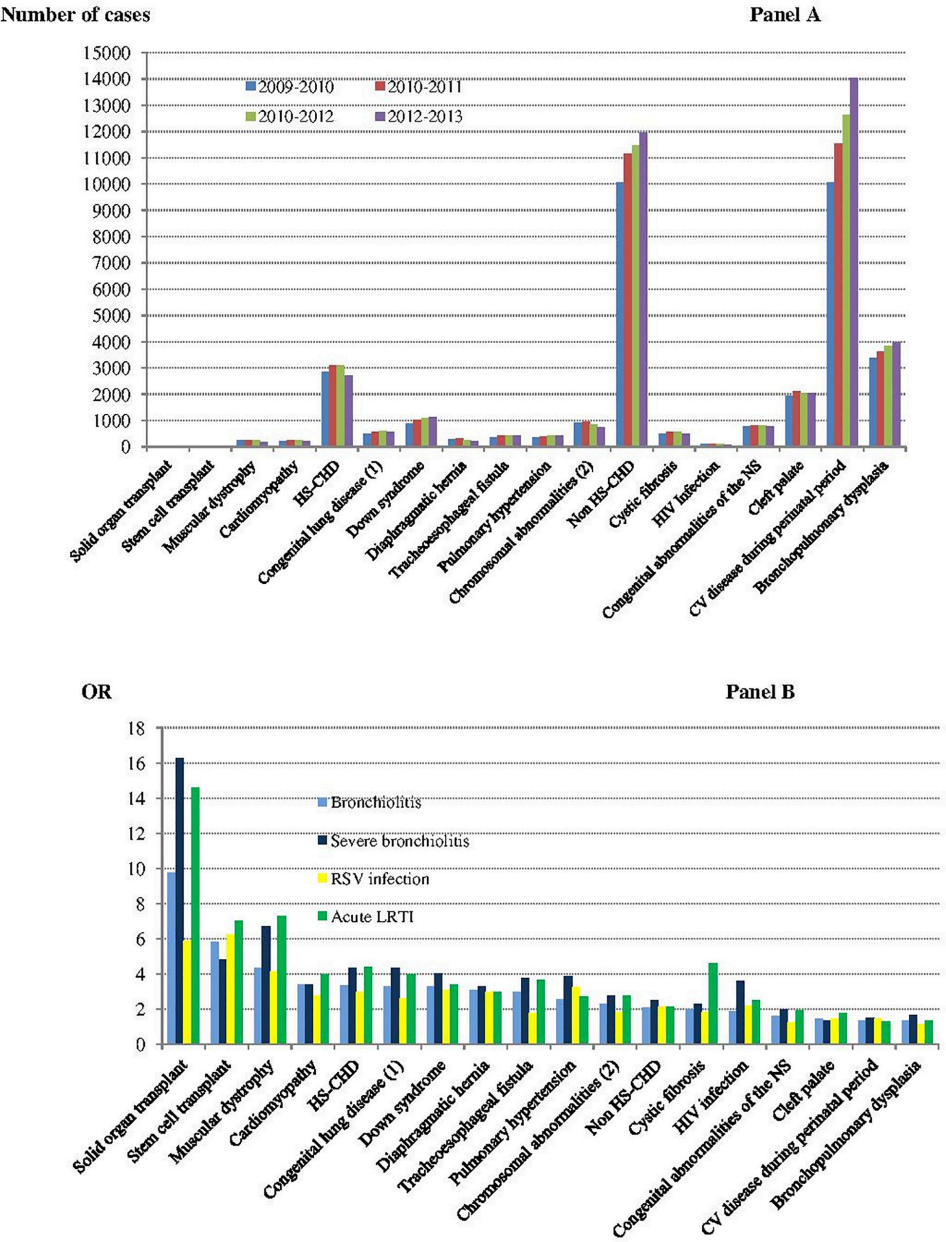
**Panel A: Number of cases with underlying medical disorders per season- Panel B: Underlying medical disorders as risk factors of hospital admission for bronchiolitis, severe bronchiolitis, RSV infection and acute LRTI (respective OR).** Abbreviations: CHD: congenital heart disease; HS: hemodynamically significant; LRTI: lower respiratory tract infection; NS: nervous system (1) Congenital lung disease and/or bronchial abnormalities (2) Other than Down syndrome.

Regarding chronic conditions under study, an increased risk of hospitalization for bronchiolitis was observed for (by decreasing order of adjusted OR): solid organ and stem cell transplants, muscular dystrophy, cardiomyopathy, HS-CHD, congenital lung disease and/or bronchial abnormalities, Down syndrome, diaphragmatic hernia, congenital tracheoesophageal fistula, pulmonary hypertension, chromosomal abnormalities other than Down syndrome, hemodynamically non-significant CHD, cystic fibrosis, HIV infection, congenital abnormalities of the nervous system, cleft palate, cardiovascular disease occurring during the perinatal period, and BPD (Table 3). Fig 2 displays number of cases with underlying medical disorders at risk per season (panel A) and related adjusted OR depending on case definition (panel B).

All associations with bronchiolitis were also found when sensitivity analyses were performed on case definition using hospitalization risk for severe bronchiolitis, RSV infection, and acute LRTI (Fig 2, panel B and S3 Table). The strength of the association with age at season onset decreased significantly with a broadened definition from RSV infection to acute LRTI. Conversely, the strength of the association of extremely preterm, BPD, and HS–CHD increased significantly with a broadened definition of bronchiolitis. Finally, all associations with bronchiolitis were also found when infants and children were selected at first bronchiolitis season exposure (S4 Table).

## Discussion

This study is the first assessing the hospitalization rates for bronchiolitis in a recent, large, nationwide cohort of all newborns and infants <2 years of age at risk over four bronchiolitis seasons. Our results show that hospitalization rates range between 1 and 1.5% (from 1.26% to 1.48%), but are significantly higher in infants with well-known risk factors such as prematurity, BPD, and HS–CHD, and those with a large range of underlying medical disorders. The risk assessment of these various medical disorders in a nationwide study represents one of the major interests of our study, in particular, with regard to treatment strategies.

The present study identifies a large range of medical disorders that are independent risk factors for bronchiolitis hospitalization (Table 3). Besides BPD and HS–CHD, these disorders comprise other cardiothoracic diseases, congenital malformations, neuromuscular diseases, chromosomal abnormalities, HIV infection, and solid organ or stem cell transplants. Although the frequency of each disorder is low, the total number of affected infants is about six-fold that of BPD and HS–CHD at each season onset (Table 1). Moreover, in most cases, the ORs for bronchiolitis hospitalization in these diseases exceed those for BPD (Table 3).

Pulmonary hypertension, congenital lung disease and/or bronchial abnormalities, and congenital tracheoesophageal fistula are conditions that are associated with a high respiratory morbidity at any age. It is thus easily understandable that these conditions are associated with an increased risk of bronchiolitis hospitalization. These infants have a reduced respiratory reserve, which explains the increased risk of hypoxemia in case of respiratory injury or aggression. RSV mortality has been noted to be higher in infants with cyanotic cardiac lesions and pulmonary hypertension than in healthy peers [21]. In pulmonary hypertension, relatively unoxygenated blood bypasses the lung and enters the systemic circulation [21]. Thus, hypoxia is already present in infants with cyanotic heart defects and/or pulmonary hypertension before RSV infection [21]. As a result, these infants are likely to have hypoxia during acute LRTI. Cystic fibrosis has been found to be a significant risk factor for RSV infection hospitalization in numerous studies [9,16]. We have recently shown that infants with congenital diaphragmatic hernia repair present diaphragmatic weakness and dysfunction during the first 2 years of life, which normalizes by 5 to 6 years of age [22]. This respiratory muscle dysfunction underlies the respiratory vulnerability of these infants during the first 2 years of life. Conversely, omphalocele was not identified as a risk factor for bronchiolitis hospitalization. Differences in the abdominal size defect and the consequent neonatal respiratory morbidity may explain why this diagnosis did not emerge as a risk factor in the present study.

Respiratory muscles are rarely spared in neuromuscular diseases. Respiratory muscle weakness and/or dysfunction may explain the increased risk of respiratory failure during acute LRTI in infants with neuromuscular disorders. Muscular dystrophy was indeed associated with a four–fold increased risk for bronchiolitis hospitalization (Table 3).

Congenital malformations of the nervous system were also associated with an increased risk for bronchiolitis hospitalization. Similar findings have been observed in other studies [9,16]. In a nationwide study in Denmark, encephalocele, spina bifida, and malformations of the spinal cord, as well as cerebral palsy, were identified as risk factors for RSV infection hospitalization [16]. Despite the fact that these diseases do not directly affect the cardiorespiratory system, ineffective cough and breathing, sleep-disordered breathing, and false passages may predispose these infants to an increased risk of bronchiolitis hospitalization.

Numerous worldwide studies have demonstrated that infants with Down syndrome are at increased risk of severe bronchiolitis and resultant morbidity, independent of concomitant CHD [9]. Infants with Down syndrome are predisposed to upper airway obstruction and obstructive sleep apnea due to altered craniofacial anatomy with midfacial and mandibular hypoplasia, glossoptosis with relative macroglossia, frequent adenotonsillar hypertrophy, muscle hypotonia with an increased prevalence of pharyngo-laryngomalacia, and subglottic and/or tracheal stenosis [23]. These anatomical predisposing factors for obstructive sleep apnea are aggravated by obesity, hypothyroidism, and gastroesophageal reflux disease [23]. Infections are associated with an increased morbidity due to immune dysfunction. All these factors explain why Down syndrome was associated with a three-fold increased risk for bronchiolitis hospitalization (Table 3).

Any type of congenital or acquired immunodepression has been shown to be associated with an increased risk for bronchiolitis hospitalization. In our study, infants with solid organ transplant or stem cell transplant had the highest OR for bronchiolitis hospitalization (Table 3). Improvements in the treatment of HIV infection may explain why this condition is a less serious risk factor for bronchiolitis hospitalization.

Regarding the conditions discussed previously, recurrent or prolonged hospital visits expose these infants to a nosocomial risk of microbiologic infection, in particular, bronchiolitis, during the winter season. This may explain, in part, the increased risk of bronchiolitis hospitalization in infants with disorders that do not affect the cardiorespiratory or immune system, such as malformations of the urinary system or the gastrointestinal tract [16]. The risk of RSV infection is a major argument for restricting hospital visits during the bronchiolitis season in these vulnerable infants.

Overall, bronchiolitis hospitalization rates found in this nationwide study in France were comparable to those observed in other West European countries [24] as well as some well-recognized risk factors for bronchiolitis hospitalization, such as male sex, multiple birth, prematurity, growth restriction, maternal smoking, and age at the onset of the RSV season [5,25,26]. BPD and HS–CHD, representing about 0.20 and 0.17% of the total population exposed each season, respectively, were also confirmed as significant risk factors for bronchiolitis hospitalization, supporting the validity of preventive strategies [6,7,27–29].

Our study has both strengths and limitations. The study is based on a nationwide sample of hospital births retrieved from the French National Hospital Discharge database, representing more than 95% of all births in France from 2008–2013 [10]. However, because of left-truncation of hospital discharge data, we could not identify hospital births before January 1, 2008, and therefore children aged 22 to 24 months at risk during the season 2009–2010. These children should represent less than 10% of all infants (Table 1) and had the lowest adjusted risk of hospitalization for bronchiolitis (Table 3) suggesting that the raw hospitalization rate for bronchiolitis may be overestimated during the season of 2009–2010 (Table 2). Also, risk factors and outcomes were identified from administrative records and may have introduced misclassification bias. Several validation studies conducted in teaching hospitals suggest that risk factors and bronchiolitis hospitalizations are correctly recorded in the French Hospital Discharge database [12,19,20]. At the national level, we identified several administrative recording problems for perinatal care that concerned a minority of hospitals and greatly improved over the study period: multiple births with the same anonymous identifier (0.79%), missing data on birth weight (0.31%), or GA (11.9%). In the main analysis, multiple births with the same anonymous identifier and infants with missing birth weight were excluded (Fig 1), and GA was imputed for missing data. Similar results were found in a complete-case analysis after excluding infants with missing data on GA. Regarding outcomes, RSV testing is not recommended in routine practice in France as in other high-income countries [19,30,31]. Reassuringly, we found that all associations with bronchiolitis hospitalization were similarly identified in sensitivity analyses with case definitions that were either more specific (severe bronchiolitis or RSV infection) or were more sensitive (acute LRTI). Moreover, our results are globally comparable to those of the nation-wide study of RSV-hospitalization in Denmark. Interestingly, we found that the influenza pandemic (H1N1) was associated with an increased risk of hospital admission in 2009–2010 for acute LRTI that did not affect other risk factors. Also, a history of bronchiolitis was strongly associated with the risk for bronchiolitis hospitalization suggesting an individual susceptibility to bronchiolitis. Finally, this is a retrospective cohort study and causation cannot be claimed.

## Conclusions

This large nationwide study confirms a list of eighteen chronic conditions that are independent risk factors for bronchiolitis hospitalization and represent approximately 2.0% of live-borns. Recognition of these disorders besides prematurity, BPD, and HS–CHD as priority targets for preventative and curative treatment strategies is warranted. Indeed, preventative and curative therapeutic options are becoming available for RSV infection and bronchiolitis. Within a global health perspective, information on updated risk factors and susceptible populations is necessary for an optimal use of new resources.

## Supporting information

S1 Appendix Literature search strategy. (DOCX)

S2 Appendix ICD–10 codes for variables. (DOCX)

S3 Appendix RECORD statement–Checklist of items, extended from the STROBE statement, for observational studies using routinely collected health data. (DOCX)

S1 Table Distribution of birth weight (grams) by gender and gestational age (source: liveborn infants discharged with complete information in metropolitan France in 2008–2013). (DOCX)

S2 Table Characteristics of infants and children <2 years at risk for bronchiolitis hospitalization over four consecutive seasons. (DOCX)

S3 Table Risk factors of hospital admission for severe bronchiolitis, RSV bronchiolitis, and acute LRTI—Sensitivity analyses (N = 7,211,751). (DOCX)

S4 Table Risk factors of hospital admission for bronchiolitis, among infants and children selected at first bronchiolitis season—Sensitivity analyses (N = 3,879,506). (DOCX)

## Abbreviations

BPD: bronchopulmonary dysplasia
CHD: congenital heart disease
CI: confidence interval
GA: gestational age
GEE: generalized Estimating Equations
HS-CHD: hemodynamically significant congenital heart disease
ICD: international Classification of Diseases
LRTI: low respiratory tract infection
NCIU: neonatal intensive care unit
OR: odds ratios
PMSI: Programme de Médicalisation des Systèmes d’Information
RSV: respiratory syncytial virus

## References

[1] Meissner HC. Viral bronchiolitis in children. N Engl J Med. 2016;374:62–72. 10.1056/NEJMra1413456 26735994

[2] Iacobelli S, Combier E, Roussot A, Cottenet J, Gouyon JB, Quantin C. Gestational age and 1-year hospital admission or mortality: a nation–wide population-based study. BMC Pediatr. 2017;17:28 10.1186/s12887-017-0787-y 28100222 PMC5242044

[3] Florin TA, Plint AC, Zorc JJ. Viral bronchiolitis. Lancet. 2017;389:211–24. 10.1016/S0140-6736(16)30951-5 27549684 PMC6765220

[4] Shi T, McAllister DA, O’Brien KL, Simoes EAF, Madhi SA, Gessner BD, et al Global, regional, and national disease burden estimates of acute lower respiratory infections due to respiratory syncytial virus in young children in 2015: a systematic review and modeling study. Lancet. 2017;390:946–58. 10.1016/S0140-6736(17)30938-8 28689664 PMC5592248

[5] Figueras–Aloy J, Manzoni P, Paes B, Simoes EAF, Bont L, Checchia PA et al Defining the risk and associated morbidity and mortality of severe respiratory syncytial virus infection among preterm infants without chronic lung disease or congenital heart disease. Infect Dis Ther. 2016;5:417–52. 10.1007/s40121-016-0130-1 27628014 PMC5125133

[6] Paes B, Fauroux B, Figueras-Aloy, Bont L, Checchia PA, Simoes EAF, et al Defining the risk and associated morbidity and mortality of severe respiratory syncytial virus infection among infants with chronic lung disease. Infect Dis Ther. 2016;5:453–71. 10.1007/s40121-016-0137-7 27864751 PMC5125140

[7] Checchia PA, Paes B, Bont L, Manzoni P, Simoes EAF, Fauroux B, et al Defining the risk and associated morbidity and mortality of severe respiratory syncytial virus infection among infants with congenital heart disease. Infect Dis Ther. 2017;37:37–56. 10.1007/s40121-016-0142-x PMC5336417 28070870

[8] Verhoeven D. Influence of immunological maturity on respiratory syncytial virus-induced morbidity in young children. Viral Immunol. 2019;32:76–83. 10.1089/vim.2018.0121 30499759

[9] Manzoni P, Figueras–Aloy J, Simões EAF, Checchia PA, Fauroux B, Bont L, et al Defining the incidence and associated morbidity and mortality of Severe Respiratory Syncytial Virus infection among children with chronic diseases. Infect Dis Ther. 2017;6:383–411. 10.1007/s40121-017-0160-3 28653300 PMC5595774

[10] INSEE. Institut national de la statistique et des études économiques. [The National Institute of Statistics and Economic Studies]. Available from: http://www.insee.fr [Accessed October 12, 2017].

[11] Agence Technique de l’Information sur l’Hospitalisation. Aide à l’utilisation des informations de chaînage [How to use de-identified patient information] Lyon, France: Agence technique de l'information sur l'hospitalisation; 2014 [updated October 6, 2015]. Available from: http://www.atih.sante.fr/aide-lutilisation-des-informations-de-chainage [Accessed October 12, 2017].

[12] Pierron A, Revert M, Goueslard K, Vuagnat A, Cottenet J, Benzenine E, et al Evaluation of the metrological quality of the medico-administrative data for perinatal indicators: A pilot study in 3 university hospitals. Rev Epidemiol Sante Publique. 2015;63:237–46. 10.1016/j.respe.2015.05.001 26143088

[13] Ancel PY, Goffinet F, Kuhn P, Langer B, Matis J, Hernandorena X, et al Survival and morbidity of preterm children born at 22 through 34 weeks' gestation in France in 2011: results of the EPIPAGE-2 cohort study. JAMA Pediatr. 2015;169:230–8. 10.1001/jamapediatrics.2014.3351 25621457

[14] Charkaluk ML, Marchand-Martin L, Ego A, Zeitlin J, Arnaud C, Burguet A, et al Epipage Study Group. The influence of fetal growth reference standards on assessment of cognitive and academic outcomes of very preterm children. J Pediatr. 2012;161:1053–8. 10.1016/j.jpeds.2012.05.037 22765954

[15] Manzoni P, Paes B, Resch B, Mejias A, Ramilo O, Carbonell-Estramy X, et al High risk for RSV bronchiolitis in late preterms and selected infants affected by rare disorders: a dilemma of specific prevention. Early Hum Dev. 2012;S34–41. 10.1016/S0378-3782(12)70012-9 22633511

[16] Kristensen K, Hjuler T, Ravn H, Smoes EA, Stensballe LG. Chronic diseases, chromosomal abnormalities, and congenital malformations as risk factors for respiratory syncytial virus hospitalization: a population-based study. Clin Infect Dis. 2012;54:810–7. 10.1093/cid/cir928 22247121

[17] Houyel L, Khoshnood B, Anderson RH, Lelong N, Thieulin AC, Goffinet F, et al Population-based evaluation of a suggested anatomic and clinical classification of congenital heart defects based on the International Paediatric and Congenital Cardiac code. Orphanet J Rare Dis. 2011;6:64 10.1186/1750-1172-6-64 21968022 PMC3198675

[18] Che D, Nicolau J, Bergounioux J, Perez T, Bitar D. Bronchiolite aiguë du nourrisson en France: bilan des cas hospitalisés en 2009 et facteurs de létalité. Arch Pediatr. 2012;19:700–6. 10.1016/j.arcped.2012.04.015 22652519

[19] ANAES. Conférence de consensus: Prise en charge de la bronchiolite du nourrisson. September 2000.

[20] Soilly AL, Ferdynus C, Desplanches O, Grimaldi M, Gouyon JB. Paediatric intensive care admissions for respiratory syncytial virus bronchiolitis in France: results of a retrospective survey and evaluation of the validity of a medical information system programme. Epidemiol Infect. 2012;140:608–16. 10.1017/S0950268811001208 21733254

[21] Welliver R. Selected populations at increased risk from RSV infection. Pediatr Infect Dis J. 2003;22: S40–5. 10.1097/01.inf.0000053884.21238.13 12671451

[22] Khirani S, Amaddeo A, Khen-Dunlop N, Olmo Arroyo J, Lapillonne A, Becquet O, et al Diaphragmatic function in infants and children with congenital diaphragmatic hernia: a cross-sectional study. Eur J Cardiothorac Surg. 2018;53:740–7. 10.1093/ejcts/ezx391 29165681

[23] Lal C, White DR, Joseph JE, van Bakergem K, LaRosa A. Sleep-disordered breathing in Down syndrome. Chest. 2015;147:570–9. 10.1378/chest.14-0266 25644910

[24] Green CA, Yeates D, Goldacre A, Sande C, Parslow RC, McShane P, et al Admission to hospital for bronchiolitis in England: trends over five decades, geographical variation and association with perinatal characteristics and subsequent asthma. Arch Dis Child. 2016;101:140–6. 10.1136/archdischild-2015-308723 26342094 PMC4752648

[25] Bont L, Checchia PA, Fauroux B, Figueras-Aloy J, Manzoni P, Paes B, et al Defining the epidemiology and burden of severe respiratory syncytial virus infection among infants and children in Western countries. Infect Dis Ther. 2016;5:271–98. 10.1007/s40121-016-0123-0 27480325 PMC5019979

[26] Stranak Z, Saliba E, Kosma P, Posfay-Barbe K, Yunis K, Farstad T, et al Predictors of RSV LRTI hospitalization in infants born at 33 to 35 weeks gestational age: a large multinational study (PONI). PloS One. 2016;11:e0157446 10.1371/journal.pone.0157446 27310438 PMC4910988

[27] American Academy of Pediatrics. Updated guidance for palivizumab prophylaxis among infants and young children at increased risk of hospitalization for respiratory syncytial virus infection. Pediatrics. 2014;134:e620–38. 10.1542/peds.2014-1666 25070304

[28] Pignotti MS, Leo MC, Pugi A, De Masi S, Biermann KP, Galli L, et al Consensus conference on the appropriateness of palivizumab prophylaxis in RSV disease. Pediatr Pulmonol. 2016;51:1088–96. 10.1002/ppul.23561 27618642

[29] Haute Autorité de Santé. Transparency Committee. December 19, 2007. Available from: https://www.has-sante.fr/portail/upload/docs/application/pdf/ct-5014_synagis.pdf [Accessed October 12, 2017].

[30] Ralston SL, Lieberthal AS, Meissner HC, Alverson BK, Baley JE, Gadomski AM, et al Clinical Practice Guideline: The Diagnosis, Management, and Prevention of Bronchiolitis. Pediatrics. 2014;134:e1474–502. 10.1542/peds.2014-2742 25349312

[31] NICE guideline. Bronchiolitis in children: diagnosis and management. June 2015. https://www.nice.org.uk/guidance/ng9/resources/bronchiolitis-in-children-diagnosis-and-management-pdf-51048523717

