# Supplementary material for: Risk factors for bronchiolitis hospitalization in infants: A French nationwide retrospective cohort study over four consecutive seasons (2009-2013)
Source: PLoS One. 2020 Mar 6;15(3):e0229766. doi: 10.1371/journal.pone.0229766 (PMC7059917; doi:10.1371/journal.pone.0229766)
Supplement: S1 Appendix — (DOCX) [file pone.0229766.s001.docx]

**Supporting information - S1 Appendix: literature search strategy**

The scope for the literature search was to investigate underlying medical disorders as risk factors for bronchiolitis hospitalizations.

We searched for articles published from January1^st^, 1995 and October 17th, 2017 using the following general terms: [((MJMESH.EXACT.EXPLODE ("Bronchiolitis, Viral")) OR (MJEMB.EXACT.EXPLODE ("viral bronchiolitis") OR MJEMB.EXACT.EXPLODE ("bronchiolitis")) OR ti,ab,su (bronchiolitis)) OR ((ti,ab,su ("lower respiratory tract infection*" OR LRTI* OR LRI* OR "lower respiratory infection*")) OR (MJEMB.EXACT.EXPLODE ("lower respiratory tract infection"))] **AND** [(MJMESH.EXACT.EXPLODE ("Hospitalization") OR (MJEMB.EXACT ("hospitalization") OR MJEMB.EXACT ("child hospitalization") OR MJEMB.EXACT ("child hospitalization") OR  (ti,ab,su (hospitaliz* or hospital N/3 admission or hospital N/3 admit* OR "ICU admi*" or "intensive care unit admi*")) **AND** [(EMB.EXACT ("risk factor")) OR (MESH.EXACT ("Risk Factors")) OR (ti,ab,su ("risk factor*" or risk*))] **AND** [ti,ab (infancy or infant* or newborn* or neonat* or "young child*" or child* or paediatr* or pediatr*)].

In addition, specific key words were used for each condition and are detailed below:

Chromosomal abnormalities: [((ti,ab,su ("chromosom* abnormalit*" OR "chromosom* disease*" OR "chromosom* disorder*")) OR (MJMESH.EXACT.EXPLODE ("Chromosome Aberrations") OR MJMESH.EXACT.EXPLODE ("Chromosome Disorders")) OR (MJEMB.EXACT.EXPLODE ("chromosome disorder") OR MJEMB.EXACT.EXPLODE ("chromosome aberration")) OR (MJEMB.EXACT.EXPLODE ("chromosome disorder"))].

Transplantation:  [(ti,ab,su (transplant or transplantation or graft or grafting)) OR (MESH.EXACT.EXPLODE ("Liver Transplantation") OR MESH.EXACT.EXPLODE ("Transplantation") OR MESH.EXACT ("Heart Transplantation") OR MESH.EXACT.EXPLODE ("Organ Transplantation")) OR (MESH.EXACT ("Lung Transplantation") OR MESH.EXACT ("Heart-Lung Transplantation")) OR (MESH.EXACT.EXPLODE ("Kidney Transplantation")) OR (MESH.EXACT ("Bone Marrow Transplantation") OR MJMESH.EXACT.EXPLODE ("Hematopoietic Stem Cell Transplantation"))  OR (EMB.EXACT.EXPLODE ("heart lung transplantation")) OR (EMB.EXACT.EXPLODE ("kidney transplantation")) OR (EMB.EXACT.EXPLODE ("liver transplantation")) OR (EMB.EXACT.EXPLODE ("lung transplantation")))]

Human immunodeficiency virus (HIV): [MJMESH.EXACT.EXPLODE ("HIV") OR (MJEMB.EXACT.EXPLODE ("acute HIV infection")) OR (MJMESH.EXACT.EXPLODE ("HIV Infections")) OR (ti,ab,su (HIV or AIDS or "human immunodeficiency virus"))]

Down syndrome: [(MJMESH.EXACT.EXPLODE ("Down Syndrome")) OR (MJEMB.EXACT.EXPLODE ("Down syndrome")) OR (ti,ab,su ("Down syndrome" or "Down's syndrome" or "trisomy 21"))]

Cardiomyopathy or cardiovascular abnormalities: [(ti(Cardiomyopath* or "cardiovascular abnormalit*")) OR (MJMESH.EXACT.EXPLODE ("Cardiomyopathy, Hypertrophic, Familial") OR MJMESH.EXACT.EXPLODE ("Cardiomyopathy, Dilated") OR MJMESH.EXACT.EXPLODE ("Cardiomyopathy, Hypertrophic") OR MJMESH.EXACT.EXPLODE("Cardiomyopathy, Restrictive")) OR (EMB.EXACT ("restrictive cardiomyopathy") OR EMB.EXACT ("nonischemic cardiomyopathy") OR EMB.EXACT ("hypertrophic obstructive cardiomyopathy") OR MJEMB.EXACT.EXPLODE ("congestive cardiomyopathy") OR EMB.EXACT ("ischemic cardiomyopathy") OR EMB.EXACT ("cardiomyopathy") OR EMB.EXACT ("familial hypertrophic cardiomyopathy") OR EMB.EXACT ("hypertrophic cardiomyopathy")) OR (MJEMB.EXACT.EXPLODE("cardiovascular malformation")) OR (MJMESH.EXACT.EXPLODE ("Cardiovascular Abnormalities"))]

Congenital malformations: [(ti,ab,su("congenital abnormalit*" or "congenital malformat*")) OR (MJMESH.EXACT.EXPLODE ("Congenital, Hereditary, and Neonatal Diseases and Abnormalities")) OR (MESH.EXACT.EXPLODE ("Congenital Abnormalities")) OR (MJEMB.EXACT.EXPLODE ("newborn disease")) OR (MJEMB.EXACT.EXPLODE ("congenital malformation"))]

Myopathy: (MESH.EXACT ("Myopathy, Central Core") OR MESH.EXACT ("Myopathies, Structural, Congenital") OR MJMESH.EXACT.EXPLODE ("Myotonia Congenita")) OR MJEMB.EXACT.EXPLODE ("myopathy") OR ti,ab,su (myopath*)

Diaphragmatic hernia: [(ti,ab,su ("diaphragmatic hernia")) OR (EMB.EXACT.EXPLODE ("diaphragm hernia")) OR (MESH.EXACT.EXPLODE ("Hernia, Diaphragmatic"))]

Cystic fibrosis: [(ti,ab,su ("cystic fibrosis")) OR (MESH.EXACT.EXPLODE ("Cystic Fibrosis")) OR (MJEMB.EXACT.EXPLODE ("cystic fibrosis"))]

Pulmonary hypertension: [(ti,ab,su ("pulmonary hypertension")) OR (MJMESH.EXACT.EXPLODE ("Familial Primary Pulmonary Hypertension") OR MJMESH.EXACT.EXPLODE ("Hypertension, Pulmonary"))]

Omphalocele: (MESH.EXACT ("Hernia, Umbilical")) OR EMB.EXACT ("omphalocele") OR ti,ab,su (omphalocele)

Cleft palate: (EMB.EXACT ("Cleft Palate") OR EMB.EXACT ("cleft palate")) OR (ti,ab,su (cleft palate)).
