# Supplementary material for: Risk factors for bronchiolitis hospitalization in infants: A French nationwide retrospective cohort study over four consecutive seasons (2009-2013)
Source: PLoS One. 2020 Mar 6;15(3):e0229766. doi: 10.1371/journal.pone.0229766 (PMC7059917; doi:10.1371/journal.pone.0229766)
Supplement: S2 Appendix — (DOCX) [file pone.0229766.s002.docx]

**Supporting information - Appendix 2: ICD–10 codes for variables**

Birth:

- Single (Z38.0, Z38.1, Z38.2)
- Twins (Z38.3, Z38.4, Z38.5)
- Multiple (Z38.6, Z38.7, Z38.8)

Intrauterine growth restriction (P05.9)

Underlying medical disorders:

- Bronchopulmonary dysplasia (BPD) originating in the perinatal period (P27.1),
- Congenital heart disease (CHD): Houyel L et al. (EPICARD Study group). *Orphanet J Rare Dis*. 2011 Oct 3;6:64
- Pulmonary hypertension (I27.0: primary pulmonary hypertension, I27.2: other secondary pulmonary hypertension)
- Congenital lung disorders and bronchial abnormalities (Q32.2: congenital bronchomalacia, Q32.3: congenital stenosis of bronchus, Q32.4: other congenital malformations of bronchus, Q34.9: congenital malformations of respiratory system, Q33: congenital malformations of lung),
- Congenital tracheoesophageal fistula (Q39.1: atresia of esophagus with tracheoesophageal fistula, Q39.2: congenital tracheoesophageal fistula without atresia), Cystic fibrosis (E84),
- Cardiovascular disease occurring during neonatal period (P29),
- Cardiomyopathy (I42: cardiomyopathy, I43: cardiomyopathy classified elsewhere),
- Diaphragmatic hernia (K44),
- Omphalocele (Q79.2),
- Muscular dystrophy (G71),
- Congenital abnormalities of the nervous system (Q00: anencephaly and similar malformations, Q03: congenital hydrocephalus, Q05: spina bifida)
- Cleft palate (Q35: cleft palate, Q37: cleft palate with unilateral cleft lip),
- Down syndrome (Q90),
- Other chromosome abnormalities (Q91, Q92, Q93, Q94, Q95, Q96, Q97, Q98, Q99·0, Q99.1, Q99.2, Q99.8),
- HIV infection (B20: HIV disease resulting in infectious and parasitic diseases, B21: HIV disease resulting in malignant neoplasms, B22: HIV disease resulting in other specified diseases, B23: HIV disease resulting in other conditions, B24: unspecified HIV disease, Z21: Asymptomatic HIV infection status).
- Solid organ transplant and stem cell transplant were identified by medical procedures.

Maternal characteristics:

- Fetus and newborn affected by maternal use of tobacco (P04.2),
- Fetus and newborn affected by other maternal circulatory and respiratory diseases (P00.3),
- Syndrome of infant of mother with gestational diabetes (P70.0) or a diabetic mother (P70.1)

Outcomes:

- Acute bronchiolitis (J21),
- RSV infection (J21.0: bronchiolitis due to RSV; J12.1: RSV pneumonia; J20.5: acute bronchitis due to RSV; LRTI (see below) and B9.74: RSV as the cause of diseases classified to other chapters),
- Acute lower respiratory tract infection (LRTI) (J09-J18: influenza and pneumonia; J2: Other acute lower respiratory infections; J85: Abscess of lung and mediastinum; P23: Congenital pneumonia)
