# Supplementary material for: Risk factors for bronchiolitis hospitalization in infants: A French nationwide retrospective cohort study over four consecutive seasons (2009-2013)
Source: PLoS One. 2020 Mar 6;15(3):e0229766. doi: 10.1371/journal.pone.0229766 (PMC7059917; doi:10.1371/journal.pone.0229766)
Supplement: S3 Appendix — (DOCX) [file pone.0229766.s003.docx]

**Supporting information**

S3 Appendix: RECORD statement – Checklist of items, extended from the STROBE statement, for observational studies using routinely collected health data

**Appendix S3. RECORD statement^1^ – checklist of items, extended from the STROBE statement, for observational studies using routinely collected health data.**

|  | **Item No.** | **STROBE items** | **Location in manuscript where items are reported** | **RECORD items** | **Location in manuscript where items are reported** |
| --- | --- | --- | --- | --- | --- |
| **Title and abstract** | | | | | |
|  | 1 | (a) Indicate the study’s design with a commonly used term in the title or the abstract (b) Provide in the abstract an informative and balanced summary of what was done and what was found | a) Done [Title]  b) Done [Abstract] | RECORD 1.1: The type of data used should be specified in the title or abstract. When possible, the name of the databases used should be included.  RECORD 1.2: If applicable, the geographic region and timeframe within which the study took place should be reported in the title or abstract.  RECORD 1.3: If linkage between databases was conducted for the study, this should be clearly stated in the title or abstract. | 1.1) Done [Abstract/Methods] The full name of the database is provided in [Methods/Data source].  1.2) Done [Abstract]  1.3) Not applicable. |
| **Introduction** | | | | | |
| Background rationale | 2 | Explain the scientific background and rationale for the investigation being reported | Done [Introduction] | | |
| Objectives | 3 | State specific objectives, including any prespecified hypotheses | Done [Introduction/last paragraph] | | |
| **Methods** | | | | | |
| Study Design | 4 | Present key elements of study design early in the paper | Done [Title; Abstract; Methods, first sentence and Study population] | | |
| Setting | 5 | Describe the setting, locations, and relevant dates, including periods of recruitment, exposure, follow-up, and data collection | Done [Title; Abstract; Methods/Study population] | | |
| Participants | 6 | *(a) Cohort study* - Give the eligibility criteria, and the sources and methods of selection of participants. Describe methods of follow-up  *Case-control study* - Give the eligibility criteria, and the sources and methods of case ascertainment and control selection. Give the rationale for the choice of cases and controls  *Cross-sectional study* - Give the eligibility criteria, and the sources and methods of selection of participants  *(b) Cohort study* - For matched studies, give matching criteria and number of exposed and unexposed  *Case-control study* - For matched studies, give matching criteria and the number of controls per case | a) Done [Methods/Study population]  Methods of follow up: Not applicable as all individuals are followed from maternity ward discharge until in-hospital or last hospital discharge in 2008-2013.  b) Not applicable. | RECORD 6.1: The methods of study population selection (such as codes or algorithms used to identify subjects) should be listed in detail. If this is not possible, an explanation should be provided.  RECORD 6.2: Any validation studies of the codes or algorithms used to select the population should be referenced. If validation was conducted for this study and not published elsewhere, detailed methods and results should be provided.  RECORD 6.3: If the study involved linkage of databases, consider use of a flow diagram or other graphical display to demonstrate the data linkage process, including the number of individuals with linked data at each stage. | 6.1) Done [Methods/Study population; Study flowchart of Figure 1]  6.2) Done [Methods/Study population].  6.3) Not applicable |
| Variables | 7 | Clearly define all outcomes, exposures, predictors, potential confounders, and effect modifiers. Give diagnostic criteria, if applicable. | Done [Methods/ Hospitalization for acute bronchiolitis and risk factors] | RECORD 7.1: A complete list of codes and algorithms used to classify exposures, outcomes, confounders, and effect modifiers should be provided. If these cannot be reported, an explanation should be provided. | A complete list of codes is provided in [Appendix S1 “ICD–10 codes for variables”.] |
| Data sources/ measurement | 8 | For each variable of interest, give sources of data and details of methods of assessment (measurement).  Describe comparability of assessment methods if there is more than one group | Done [Methods/Data source] & [Appendix S1 “ICD–10 codes for variables”]  Not applicable. | | |
| Bias | 9 | Describe any efforts to address potential sources of bias | Done with sensitivity analyses on case definition and modeling of repeated measurements [Methods/Statistical analysis]. | | |
| Study size | 10 | Explain how the study size was arrived at | Not applicable: the study included all newborns discharged alive in metropolitan France as identified in the French National Hospital Discharge database [Methods/Study population]. | | |
| Quantitative variables | 11 | Explain how quantitative variables were handled in the analyses. If applicable, describe which groupings were chosen, and why | Not applicable | | |
| Statistical methods | 12 | (a) Describe all statistical methods, including those used to control for confounding  (b) Describe any methods used to examine subgroups and interactions  (c) Explain how missing data were addressed  (d) *Cohort study* - If applicable, explain how loss to follow-up was addressed  *Case-control study* - If applicable, explain how matching of cases and controls was addressed  *Cross-sectional study* - If applicable, describe analytical methods taking account of sampling strategy  (e) Describe any sensitivity analyses | a) Done [Methods/Statistical analysis]  b) Done with interactions [Methods/ Statistical analyses; Discussion/Limitations].  c) Done. Missing data on gestational age were imputed [Methods/ Hospitalization for acute bronchiolitis and risk factors; Discussion/Limitations].  d) Not applicable as all individuals are followed until in-hospital or last hospital discharge in 2008-2013 [Methods/ Statistical analyses, first paragraph].  e) Multiple sensitivity analyses were conducted on case definition and modeling of repeated measurements. All results of sensitivity analyses are provided in [Supplementary Material: Tables S3-S4]. | | |
| Data access and cleaning methods |  | .. |  | RECORD 12.1: Authors should describe the extent to which the investigators had access to the database population used to create the study population.  RECORD 12.2: Authors should provide information on the data cleaning methods used in the study. | 12.1) Done with full access to the database population [Methods/Data source]  12.2) Data cleaning was limited as outcomes and risk factors were principally based on discharge diagnosis ICD-10 codes. |
| Linkage |  | .. |  | RECORD 12.3: State whether the study included person-level, institutional-level, or other data linkage across two or more databases. The methods of linkage and methods of linkage quality evaluation should be provided. | The study additionally included linkage on postal codes of residency to control for environmental factors of the infants and children [Methods/Confounding factors]. Postal codes of residency (n=5,645) were linked with indicators based on the PMSI database or French census data (INSEE, January 2011). |
| **Results** | | | | | |
| Participants | 13 | (a) Report the numbers of individuals at each stage of the study (*e.g.*, numbers potentially eligible, examined for eligibility, confirmed eligible, included in the study, completing follow-up, and analysed)  (b) Give reasons for non-participation at each stage.  (c) Consider use of a flow diagram | a) Done [Figure 1: Study flowchart]  b) Not applicable  c) Done [Figure 1: Study flowchart | RECORD 13.1: Describe in detail the selection of the persons included in the study (*i.e.,* study population selection) including filtering based on data quality, data availability and linkage. The selection of included persons can be described in the text and/or by means of the study flow diagram. | Done [Methods/Study population & Figure 1: Study flowchart] |
| Descriptive data | 14 | (a) Give characteristics of study participants (*e.g.*, demographic, clinical, social) and information on exposures and potential confounders  (b) Indicate the number of participants with missing data for each variable of interest  (c) *Cohort study* - summarise follow-up time (*e.g.*, average and total amount) | a) Done [Table 1].  b) Done for gestational age [Methods/ Hospitalization for acute bronchiolitis and risk factors].  c) Not applicable: all selected individuals are exhaustively followed from maternity ward discharge until in-hospital death or last discharge from 2008 to 2013. | | |
| Outcome data | 15 | *Cohort study* - Report numbers of outcome events or summary measures over time  *Case-control study* - Report numbers in each exposure category, or summary measures of exposure  *Cross-sectional study* - Report numbers of outcome events or summary measures | Done [Table 2]. | | |
| Main results | 16 | (a) Give unadjusted estimates and, if applicable, confounder-adjusted estimates and their precision (e.g., 95% confidence interval). Make clear which confounders were adjusted for and why they were included  (b) Report category boundaries when continuous variables were categorized  (c) If relevant, consider translating estimates of relative risk into absolute risk for a meaningful time period | a) Done with unadjusted estimates [Supplementary Material: Table S2].  No variable selection was performed in multivariate analysis [Statistical analysis, first paragraph] and was restated in the results [Table 3 footnote].  b) Done with gestational age and growth deficiency [Methods/ Hospitalization for acute bronchiolitis and risk factors].  c) Not relevant. | | |
| Other analyses | 17 | Report other analyses done—e.g., analyses of subgroups and interactions, and sensitivity analyses | All results of sensitivity analyses are provided in [Supplementary Material: Tables S3 et S4]. | | |
| **Discussion** | | | | | |
| Key results | 18 | Summarise key results with reference to study objectives | Done [Discussion, first paragraph]. | | |
| Limitations | 19 | Discuss limitations of the study, taking into account sources of potential bias or imprecision. Discuss both direction and magnitude of any potential bias | Done [Discussion/limitations] | RECORD 19.1: Discuss the implications of using data that were not created or collected to answer the specific research question(s). Include discussion of misclassification bias, unmeasured confounding, missing data, and changing eligibility over time, as they pertain to the study being reported. | Done [Discussion/limitations] |
| Interpretation | 20 | Give a cautious overall interpretation of results considering objectives, limitations, multiplicity of analyses, results from similar studies, and other relevant evidence | Done [Discussion] | | |
| Generalisability | 21 | Discuss the generalisability (external validity) of the study results | Done [Discussion] | | |
| **Other Information** | | | | | |
| Funding | 22 | Give the source of funding and the role of the funders for the present study and, if applicable, for the original study on which the present article is based | Done [Abstract; Methods/ Role of the funding source]. | | |
| Accessibility of protocol, raw data, and programming code |  |  |  | RECORD 22.1: Authors should provide information on how to access any supplemental information such as the study protocol, raw data, or programming code. | Raw data of the French National Hospital Discharge database cannot be shared without due permission from Health Authorities. |

**Reference Appendix S3:**

1. Benchimol EI, Smeeth L, Guttmann A, Harron K, Moher D, Petersen I, Sørensen HT, von Elm E, Langan SM, the RECORD Working Committee. The REporting of studies Conducted using Observational Routinely-collected health Data (RECORD) Statement. *PLoS Medicine* 2015;12(10):e1001885..
