# Supplementary material for: Risk factors for bronchiolitis hospitalization in infants: A French nationwide retrospective cohort study over four consecutive seasons (2009-2013)
Source: PLoS One. 2020 Mar 6;15(3):e0229766. doi: 10.1371/journal.pone.0229766 (PMC7059917; doi:10.1371/journal.pone.0229766)
Supplement: S1 Table — (DOCX) [file pone.0229766.s004.docx]

|  |  | **S1 TABLE. Distribution of birth weight (grams) by gender and gestational age (source: liveborn infants discharged with complete information in metropolitan France in 2008-2013)** | | | | | | | | | | | | | | | | | | | | | |  |
| --- | --- | --- | --- | --- | --- | --- | --- | --- | --- | --- | --- | --- | --- | --- | --- | --- | --- | --- | --- | --- | --- | --- | --- | --- |
|  |  |  | |  | **Female sex** | | | | | | | | |  | **Male sex** | | | | | | | | |  |
|  |  | **Gestational age (weeks)** | |  | **Live births** | **p5*** | **p10*** | **Q1** |  | **Median** | **Q3** | **p90** | **p95** |  | **Live births** | **p5*** | **p10*** | **Q1** |  | **Median** | **Q3** | **p90** | **p95** |  |
|  |  | **Extremely preterm (22–27 GA)** | **22** |  | 151 | 380 | 405 | 430 |  | 480 | 520 | 580 | 660 |  | 192 | 399 | 410 | 475 |  | 510 | 552 | 620 | 720 |  |
|  |  |  | **23** |  | 208 | 400 | 440 | 500 |  | 560 | 600 | 640 | 710 |  | 287 | 460 | 500 | 540 |  | 590 | 640 | 690 | 730 |  |
|  |  |  | **24** |  | 549 | 500 | 520 | 590 |  | 650 | 700 | 750 | 780 |  | 618 | 500 | 560 | 620 |  | 680 | 740 | 785 | 810 |  |
|  |  |  | **25** |  | 928 | 500 | 550 | 640 |  | 720 | 785 | 840 | 880 |  | 1,041 | 545 | 600 | 700 |  | 780 | 840 | 890 | 940 |  |
|  |  |  | **26** |  | 1228 | 530 | 590 | 700 |  | 800 | 890 | 957 | 1000 |  | 1418 | 570 | 635 | 760 |  | 870 | 960 | 1025 | 1080 |  |
|  |  |  | **27** |  | 1545 | 550 | 610 | 750 |  | 900 | 1000 | 1110 | 1175 |  | 1814 | 630 | 700 | 830 |  | 984 | 1085 | 1175 | 1239 |  |
|  |  | **Very preterm  (28–32 GA)** | **28** |  | 1831 | 650 | 720 | 850 |  | 1010 | 1140 | 1250 | 1320 |  | 2180 | 680 | 752 | 920 |  | 1100 | 1230 | 1340 | 1410 |  |
|  |  |  | **29** |  | 2198 | 750 | 840 | 990 |  | 1150 | 1300 | 1440 | 1505 |  | 2601 | 790 | 890 | 1060 |  | 1249 | 1400 | 1530 | 1620 |  |
|  |  |  | **30** |  | 2902 | 840 | 930 | 1100 |  | 1300 | 1470 | 1610 | 1700 |  | 3240 | 900 | 1000 | 1200 |  | 1420 | 1580 | 1720 | 1830 |  |
|  |  |  | **31** |  | 3834 | 980 | 1080 | 1265 |  | 1480 | 1650 | 1800 | 1900 |  | 4377 | 1020 | 1150 | 1370 |  | 1590 | 1775 | 1940 | 2050 |  |
|  |  |  | **32** |  | 5564 | 1110 | 1220 | 1430 |  | 1670 | 1860 | 2035 | 2160 |  | 6616 | 1195 | 1300 | 1540 |  | 1790 | 1985 | 2160 | 2280 |  |
|  |  | **Moderate preterm (33–36 GA)** | **33** |  | 8356 | 1270 | 1410 | 1650 |  | 1885 | 2095 | 2300 | 2440 |  | 9956 | 1350 | 1500 | 1770 |  | 2000 | 2200 | 2400 | 2530 |  |
|  |  |  | **34** |  | 14679 | 1500 | 1640 | 1880 |  | 2110 | 2340 | 2560 | 2714 |  | 17462 | 1560 | 1720 | 1992 |  | 2230 | 2450 | 2670 | 2820 |  |
|  |  |  | **35** |  | 25248 | 1710 | 1860 | 2110 |  | 2365 | 2620 | 2880 | 3070 |  | 29562 | 1800 | 1960 | 2220 |  | 2485 | 2740 | 2995 | 3180 |  |
|  |  |  | **36** |  | 49334 | 1930 | 2090 | 2350 |  | 2610 | 2880 | 3140 | 3340 |  | 57187 | 2020 | 2200 | 2465 |  | 2730 | 2990 | 3250 | 3440 |  |
|  |  | **Full-term  (37–41 GA)** | **37** |  | 116916 | 2170 | 2330 | 2580 |  | 2850 | 3120 | 3400 | 3580 |  | 133380 | 2280 | 2450 | 2705 |  | 2980 | 3250 | 3525 | 3710 |  |
|  |  |  | **38** |  | 304134 | 2425 | 2570 | 2810 |  | 3060 | 3340 | 3610 | 3780 |  | 330402 | 2540 | 2690 | 2930 |  | 3200 | 3480 | 3750 | 3930 |  |
|  |  |  | **39** |  | 545917 | 2610 | 2740 | 2970 |  | 3212 | 3480 | 3740 | 3910 |  | 565227 | 2720 | 2860 | 3095 |  | 3360 | 3635 | 3900 | 4070 |  |
|  |  |  | **40** |  | 551930 | 2740 | 2870 | 3095 |  | 3350 | 3620 | 3880 | 4040 |  | 550240 | 2860 | 3000 | 3230 |  | 3500 | 3770 | 4040 | 4210 |  |
|  |  |  | **41** |  | 342198 | 2840 | 2980 | 3200 |  | 3470 | 3750 | 4020 | 4190 |  | 351713 | 2970 | 3100 | 3350 |  | 3630 | 3920 | 4190 | 4365 |  |
|  |  | **Post-term**  **(≥42 GA)** | **42** |  | 15254 | 2860 | 3000 | 3240 |  | 3520 | 3815 | 4080 | 4250 |  | 16722 | 3000 | 3160 | 3410 |  | 3690 | 3985 | 4260 | 4430 |  |
|  |  |  | **43** |  | 553 | 2745 | 2875 | 3140 |  | 3410 | 3740 | 4025 | 4140 |  | 599 | 2840 | 3010 | 3260 |  | 3540 | 3860 | 4190 | 4400 |  |
|  |  |  | **≥44** |  | 262 | 2450 | 2720 | 3000 |  | 3410 | 3650 | 3895 | 3960 |  | 256 | 2620 | 2945 | 3250 |  | 3510 | 3860 | 4120 | 4430 |  |
