# Supplementary material for: Risk factors for bronchiolitis hospitalization in infants: A French nationwide retrospective cohort study over four consecutive seasons (2009-2013)
Source: PLoS One. 2020 Mar 6;15(3):e0229766. doi: 10.1371/journal.pone.0229766 (PMC7059917; doi:10.1371/journal.pone.0229766)
Supplement: S2 Table — (DOCX) [file pone.0229766.s005.docx]

|  | **S2 TABLE. Characteristics of infants and children <2 years at risk for bronchiolitis hospitalization over four consecutive seasons** | | | | | | | | | | | | | | |  |
| --- | --- | --- | --- | --- | --- | --- | --- | --- | --- | --- | --- | --- | --- | --- | --- | --- |
|  |  |  | **2009-2010** | |  | **2010-2011** | |  | **2011-2012** | |  | **2012-2013** | |  |  |  |
|  | **Characteristics, n (%)** |  | **At-risk cohort** | **Bronchiolitis hospitalization** |  | **At-risk cohort** | **Bronchiolitis hospitalization** |  | **At-risk cohort** | **Bronchiolitis hospitalization** |  | **At-risk cohort** | **Bronchiolitis hospitalization** |  | **P-value^d^** |  |
|  |  |  | 1,635,123 | 23,466 (1.44) |  | 1,848,779 | 23,218 (1.26) |  | 1,868,665 | 25,352 (1.36) |  | 1,859,004 | 27,458 (1.48) |  |  |  |
|  | **Characteristics of the infant and delivery:** |  |  |  |  |  |  |  |  |  |  |  |  |  |  |  |
|  | Male sex |  | 837,913 (51.24) | 13,468 (1.61) |  | 945,688 (51.15) | 13,309 (1.41) |  | 954,386 (51.07) | 14,569 (1.53) |  | 948,580 (51.03) | 15,675 (1.65) |  | .89 |  |
|  | Multiple birth |  | 22,676 (1.39) | 661 (2.91) |  | 25,726 (1.39) | 669 (2.60) |  | 26,517 (1.42) | 761 (2.87) |  | 26,495 (1.43) | 785 (2.96) |  | .75 |  |
|  | Gestational age: |  |  |  |  |  |  |  |  |  |  |  |  |  |  |  |
|  | Extremely preterm (22–27 GA) |  | 2,193 (0.13) | 203 (9.26) |  | 2,400 (0.13) | 207 (8.63) |  | 2,485 (0.13) | 234 (9.42) |  | 2,495 (0.13) | 259 (10.38) |  | .79 |  |
|  | Very preterm (28–32 GA) |  | 13,015 (0.80) | 790 (6.07) |  | 14,281 (0.77) | 798 (5.59) |  | 14,639 (0.78) | 892 (6.09) |  | 14,572 (0.78) | 833 (5.72) |  | .008 |  |
|  | Moderate preterm (33–36 GA) |  | 81,220 (4.97) | 2,367 (2.91) |  | 93,526 (5.06) | 2446 (2.62) |  | 95,515 (5.11) | 2572 (2.69) |  | 96,174 (5.17) | 2,743 (2.85) |  | .038 |  |
|  | Full-term (≥37 GA) |  | 1,538,695 (94.10) | 20,106 (1.31) |  | 1,738,572 (94.04) | 19,767 (1.14) |  | 1,756,026 (93.97) | 21,654 (1.23) |  | 1,745,763 (93.91) | 23,623 (1.35) |  | <.0001 |  |
|  | Growth restriction: |  |  |  |  |  |  |  |  |  |  |  |  |  |  |  |
|  | Intrauterine growth restriction |  | 6,135 (0.38) | 172 (2.80) |  | 7,339 (0.40) | 163 (2.22) |  | 7,352 (0.39) | 200 (2.72) |  | 7,107 (0.38) | 230 (3.24) |  | .19 |  |
|  | Very small for GA (birth weight <5%) |  | 55,060 (3.37) | 1,151 (2.09) |  | 81,090 (4.39) | 1,350 (1.66) |  | 88,934 (4.76) | 1,417 (1.59) |  | 88,980 (4.79) | 1,480 (1.66) |  | <.0001 |  |
|  | Small for GA (birth weight: 5–10%) |  | 55,652 (3.40) | 997 (1.79) |  | 81,592 (4.41) | 1,189 (1.46) |  | 89,506 (4.79) | 1,268 (1.42) |  | 89,755 (4.83) | 1,421 (1.58) |  | <.0001 |  |
|  | Time elapsed from neonatal discharge to October 1: |  |  |  |  |  |  |  |  |  |  |  |  |  |  |  |
|  | >18 months |  | 173,282 (10.60) | 316 (0.18) |  | 348,781 (18.87) | 471 (0.14) |  | 366,043 (19.59) | 652 (0.18) |  | 371,491 (19.98) | 604 (0.16) |  | <.0001 |  |
|  | 13–18 months |  | 364,140 (22.27) | 1,152 (0.32) |  | 374,575 (20.26) | 1,216 (0.32) |  | 379,513 (20.31) | 1,398 (0.37) |  | 379,723 (20.43) | 1,395 (0.37) |  | <.0001 |  |
|  | 7–12 months |  | 352,397 (21.55) | 2,854 (0.81) |  | 369,573 (19.99) | 2,829 (0.77) |  | 375,368 (20.09) | 3,247 (0.87) |  | 366,998 (19.74) | 3,342 (0.91) |  | <.0001 |  |
|  | 3–6 months (April–June) |  | 181,082 (11.07) | 2,835 (1.57) |  | 185,080 (10.01) | 2,735 (1.48) |  | 184,839 (9.89) | 3,321 (1.80) |  | 181,969 (9.79) | 3,475 (1.91) |  | <.0001 |  |
|  | 1–3 months (July–September) |  | 193,695 (11.85) | 5,295 (2.73) |  | 194,679 (10.53) | 5,183 (2.66) |  | 195,182 (10.44) | 6,444 (3.30) |  | 192,119 (10.33) | 7,368 (3.84) |  | <.0001 |  |
|  | RSV season onset (October–December) |  | 187,132 (11.44) | 8,423 (4.50) |  | 195,191 (10.56) | 8,375 (4.29) |  | 187,144 (10.01) | 8,789 (4.70) |  | 190,253 (10.23) | 10,102 (5.31) |  | <.0001 |  |
|  | RSV season end (January–March) |  | 183,395 (11.22) | 2,591 (1.41) |  | 180,900 (9.78) | 2,409 (1.33) |  | 180,576 (9.66) | 1,501 (0.83) |  | 176,451 (9.49) | 1,172 (0.66) |  | <.0001 |  |
|  | Discharge from NICU (October–December) |  | 8,194 (0.50) | 795 (9.70) |  | 8,727 (0.47) | 812 (9.30) |  | 8,415 (0.45) | 848 (10.08) |  | 8,654 (0.47) | 922 (10.65) |  | .23 |  |
|  | History of severe respiratory infection: |  |  |  |  |  |  |  |  |  |  |  |  |  |  |  |
|  | Bronchiolitis |  | 25,091 (1.53) | 766 (3.05) |  | 36,392 (1.97) | 906 (2.49) |  | 39,515 (2.11) | 1,072 (2.71) |  | 41,234 (2.22) | 1,055 (2.56) |  | <.0001 |  |
|  | Other LRTI |  | 6,142 (0.38) | 215 (3.50) |  | 8,592 (0.46) | 207 (2.41) |  | 7,499 (0.40) | 242 (3.23) |  | 7,219 (0.39) | 212 (2.94) |  | .017 |  |
|  | Underlying medical disorders: |  |  |  |  |  |  |  |  |  |  |  |  |  |  |  |
|  | Bronchopulmonary dysplasia^b^ |  | 3,375 (0.21) | 355 (10.52) |  | 3,619 (0.20) | 350 (9.67) |  | 3,825 (0.20) | 415 (10.85) |  | 3,992 (0.21) | 388 (9.72) |  | .036 |  |
|  | Congenital heart disease^c^ |  |  |  |  |  |  |  |  |  |  |  |  |  |  |  |
|  | Hemodynamically significant CHD (surgery)^c^ |  | 2,856 (0.17) | 217 (7.60) |  | 3,096 (0.17) | 238 (7.69) |  | 3,103 (0.17) | 250 (8.06) |  | 2,721 (0.15) | 221 (8.12) |  | .39 |  |
|  | Hemodynamically non-significant CHD^c^ |  | 10,054 (0.61) | 629 (6.26) |  | 11,140 (0.60) | 558 (5.01) |  | 11,460 (0.61) | 636 (5.55) |  | 11,949 (0.64) | 778 (6.51) |  | .13 |  |
|  | Other underlying medical disorders^b^ |  | 16,818 (1.03) | 633 (3.76) |  | 18,918 (1.02) | 662 (3.50) |  | 19,836 (1.06) | 777 (3.92) |  | 20,934 (1.13) | 828 (3.96) |  | .26 |  |
|  | Pulmonary hypertension |  | 363 (0.02) | 24 (6.61) |  | 377 (0.02) | 21 (5.57) |  | 414 (0.02) | 35 (8.45) |  | 412 (0.02) | 29 (7.04) |  | .50 |  |
|  | Congenital lung disease and/or bronchial abnormalities |  | 497 (0.03) | 28 (5.63) |  | 573 (0.03) | 38 (6.63) |  | 592 (0.03) | 41 (6.93) |  | 566 (0.03) | 39 (6.89) |  | .63 |  |
|  | Congenital tracheoesophageal fistula |  | 343 (0.02) | 37 (10.79) |  | 406 (0.02) | 46 (11.33) |  | 408 (0.02) | 41 (10.05) |  | 412 (0.02) | 46 (11.17) |  | .76 |  |
|  | Cystic fibrosis |  | 481 (0.03) | 24 (4.99) |  | 550 (0.03) | 29 (5.27) |  | 551 (0.03) | 21 (3.81) |  | 489 (0.03) | 26 (5.32) |  | .56 |  |
|  | Cardiovascular disease occurring during the perinatal period without CHD identified in the follow-up |  | 10,054 (0.61) | 279 (2.78) |  | 11,542 (0.62) | 279 (2.42) |  | 12,614 (0.68) | 339 (2.69) |  | 14,037 (0.76) | 408 (2.91) |  | .98 |  |
|  | Cardiomyopathy |  | 211 (0.01) | 20 (9.48) |  | 233 (0.01) | 15 (6.44) |  | 242 (0.01) | 17 (7.02) |  | 224 (0.01) | 16 (7.14) |  | .76 |  |
|  | Diaphragmatic hernia |  | 295 (0.02) | 19 (6.44) |  | 302 (0.02) | 16 (5.30) |  | 261 (0.01) | 20 (7.66) |  | 211 (0.01) | 17 (8.06) |  | .75 |  |
|  | Omphalocele |  | 264 (0.02) | 8 (3.03) |  | 258 (0.01) | 9 (3.49) |  | 237 (0.01) | 9 (3.80) |  | 274 (0.01) | 6 (2.19) |  | .58 |  |
|  | Muscular dystrophy |  | 250 (0.02) | 18 (7.20) |  | 246 (0.01) | 21 (8.54) |  | 238 (0.01) | 27 (11.34) |  | 178 (0.01) | 15 (8.43) |  | .32 |  |
|  | Congenital abnormalities of the nervous system |  | 761 (0.05) | 23 (3.02) |  | 822 (0.04) | 29 (3.53) |  | 793 (0.04) | 43 (5.42) |  | 768 (0.04) | 36 (4.69) |  | .074 |  |
|  | Cleft palate |  | 1,925 (0.12) | 52 (2.70) |  | 2,117 (0.11) | 53 (2.50) |  | 2,059 (0.11) | 72 (3.50) |  | 2,027 (0.11) | 61 (3.01) |  | .27 |  |
|  | Down syndrome |  | 876 (0.05) | 95 (10.84) |  | 1,029 (0.06) | 77 (7.48) |  | 1,109 (0.06) | 108 (9.74) |  | 1,120 (0.06) | 118 (10.54) |  | .36 |  |
|  | Other chromosomal abnormality |  | 924 (0.06) | 56 (6.06) |  | 952 (0.05) | 65 (6.83) |  | 840 (0.04) | 47 (5.60) |  | 725 (0.04) | 72 (9.93) |  | .013 |  |
|  | HIV infection |  | 111 (0.01) | 3 (2.70) |  | 116 (0.01) | 3 (2.59) |  | 101 (0.01) | 4 (3.96) |  | 74 (0.00) | 3 (4.05) |  | .92 |  |
|  | Solid organ transplant |  | 21 (0.00) | 3 (14.29) |  | 23 (0.00) | 1 (4.35) |  | 27 (0.00) | 4 (14.81) |  | 26 (0.00) | 4 (15.38) |  | .69 |  |
|  | Stem cell transplant |  | 43 (0.00) | 5 (11.63) |  | 45 (0.00) | 3 (6.67) |  | 44 (0.00) | 5 (11.36) |  | 49 (0.00) | 3 (6.12) |  | .69 |  |
|  | Maternal disorders during pregnancy: |  |  |  |  |  |  |  |  |  |  |  |  |  |  |  |
|  | Maternal smoking |  | 31,426 (1.92) | 749 (2.38) |  | 37,752 (2.04) | 867 (2.30) |  | 39,745 (2.13) | 890 (2.24) |  | 42,559 (2.29) | 985 (2.31) |  | .009 |  |
|  | Cardiovascular or respiratory diseases |  | 1,134 (0.07) | 27 (2.38) |  | 1,255 (0.07) | 22 (1.75) |  | 1,269 (0.07) | 27 (2.13) |  | 1,264 (0.07) | 30 (2.37) |  | .94 |  |
|  | Diabetes mellitus |  | 31,647 (1.94) | 607 (1.92) |  | 40,497 (2.19) | 671 (1.66) |  | 45,558 (2.44) | 748 (1.64) |  | 49,635 (2.67) | 988 (1.99) |  | .11 |  |
|  | Abbreviations: CHD, congenital heart disease; GA, gestational age; ICD–10, 10^th^ revised edition of the International Classification of Diseases; LRTI, lower respiratory tract infection; NICU, neonatal intensive care unit; RSV, respiratory syncytial virus.  ^a^Cohort population at October 1. Each newborn was followed over two consecutive bronchiolitis seasons (except death before October 1).  ^b^ICD–10 codes were identified from hospital records.  ^c^CHD was identified according to the criteria from the Anatomic and Clinical Classification of Congenital Heart Disease and corresponding to validated ICD–10 codes. Cardiac surgery was identified as hemodynamically significant CHD.  ^d^Breslow-Day test for homogeneity of the odds-ratios across the four bronchiolitis seasons. | | | | | | | | | | | | | | |  |
|  |  |  |  |  |  |  |  |  |  |  |  |  |  |  |  |  |
