# Supplementary material for: Risk factors for bronchiolitis hospitalization in infants: A French nationwide retrospective cohort study over four consecutive seasons (2009-2013)
Source: PLoS One. 2020 Mar 6;15(3):e0229766. doi: 10.1371/journal.pone.0229766 (PMC7059917; doi:10.1371/journal.pone.0229766)
Supplement: S3 Table — (DOCX) [file pone.0229766.s006.docx]

|  | **S3 TABLE. Risk factors of hospital admission for severe bronchiolitis, RSV infection, and acute LRTI – Sensitivity analyses (N=7,211,751)** | | | | | | | | | |  |
| --- | --- | --- | --- | --- | --- | --- | --- | --- | --- | --- | --- |
|  | Variables |  | **Severe bronchiolitis  (n=27,883)** | **P-value** |  | **RSV infection**  **(n=50,515)** | **P-value** |  | **Acute LRTI**  **(n=121,969)** | **P-value** |  |
|  | **Epidemics (ref: 2011–2012):** |  |  |  |  |  |  |  |  |  |  |
|  | 2009–2010 |  | 0.84 (0.81–0.87) | <.0001 |  | 0.99 (0.97–1.02) | .63 |  | 1.02 (1.00–1.04) | .005 |  |
|  | 2010–2011 |  | 0.85 (0.82–0.89) | <.0001 |  | 0.91 (0.89–0.94) | <.0001 |  | 0.93 (0.91–0.94) | <.0001 |  |
|  | 2012–2013 |  | 1.13 (1.09–1.17) | <.0001 |  | 1.10 (1.07–1.13) | <.0001 |  | 1.08 (1.06–1.10) | <.0001 |  |
|  | **Characteristics of the infant and delivery:** |  |  |  |  |  |  |  |  |  |  |
|  | Male sex |  | 1.17 (1.14–1.20) | <.0001 |  | 1.16 (1.13–1.18) | <.0001 |  | 1.26 (1.25–1.28) | <.0001 |  |
|  | Multiple birth |  | 1.21 (1.11–1.30) | <.0001 |  | 1.27 (1.20–1.35) | <.0001 |  | 1.21 (1.16–1.26) | <.0001 |  |
|  | Gestational age (ref: ≥37 GA): |  |  |  |  |  |  |  |  |  |  |
|  | Extremely preterm (22–27 GA) |  | 2.41 (2.00–2.91) | <.0001 |  | 1.87 (1.57–2.23) | <.0001 |  | 2.39 (2.15–2.66) | <.0001 |  |
|  | Very preterm (28–32 GA) |  | 3.02 (2.76–3.30) | <.0001 |  | 2.51 (2.33–2.70) | <.0001 |  | 2.94 (2.81–3.07) | <.0001 |  |
|  | Moderate preterm (33–36 GA) |  | 2.23 (2.13–2.33) | <.0001 |  | 1.92 (1.86–1.99) | <.0001 |  | 1.88 (1.84–1.92) | <.0001 |  |
|  | Growth restriction: |  |  |  |  |  |  |  |  |  |  |
|  | Intrauterine growth restriction |  | 1.21 (1.04–1.40) | .033 |  | 1.26 (1.12–1.42) | <.0001 |  | 1.34 (1.24–1.44) | <.0001 |  |
|  | Very small for GA (birth weight <5%) |  | 1.14 (1.07–1.21) | <.001 |  | 1.09 (1.04–1.14) | <.0001 |  | 1.14 (1.11–1.17) | <.0001 |  |
|  | Small for GA (birth weight: 5–10%) |  | 1.01 (0.95–1.07) | .80 |  | 1.05 (1.01–1.10) | .025 |  | 1.08 (1.05–1.11) | <.0001 |  |
|  | Time elapsed from neonatal discharge to October 1 (ref: 7–12 months): |  |  |  |  |  |  |  |  |  |  |
|  | >18 months |  | 0.19 (0.17–0.21) | <.0001 |  | 0.21 (0.20–0.23) | <.0001 |  | 0.35 (0.34–0.37) | <.0001 |  |
|  | 13–18 months |  | 0.40 (0.37–0.44) | <.0001 |  | 0.41 (0.39–0.43) | <.0001 |  | 0.55 (0.54–0.57) | <.0001 |  |
|  | 3–6 months (April–June) |  | 2.24 (2.11–2.38) | <.0001 |  | 2.47 (2.36–2.58) | <.0001 |  | 1.92 (1.87–1.96) | <.0001 |  |
|  | 1–3 months (July–September) |  | 4.69 (4.46–4.94) | <.0001 |  | 5.48 (5.28–5.69) | <.0001 |  | 3.36 (3.29–3.43) | <.0001 |  |
|  | RSV season onset (October–December) |  | 9.55 (9.10–10.01) | <.0001 |  | 9.85 (9.50–10.20) | <.0001 |  | 4.90 (4.80–5.00) | <.0001 |  |
|  | RSV season end (January–March) |  | 2.41 (2.27–2.55) | <.0001 |  | 1.99 (1.90–2.08) | <.0001 |  | 1.11 (1.08–1.14) | <.0001 |  |
|  | Discharge from NICU (October–December) |  | 1.18 (1.09–1.26) | <.0001 |  | 1.06 (1.00–1.13) | .057 |  | 1.18 (1.13–1.23) | <.0001 |  |
|  | History of respiratory infection: |  |  |  |  |  |  |  |  |  |  |
|  | Bronchiolitis |  | 3.34 (3.05–3.66) | <.0001 |  | 3.97 (3.73–4.23) | <.0001 |  | 3.88 (3.74–4.01) | <.0001 |  |
|  | Other LRTI |  | 2.09 (1.78–2.46) | <.0001 |  | 2.75 (2.42–3.12) | <.0001 |  | 3.46 (3.23–3.72) | <.0001 |  |
|  | Underlying medical disorders: |  |  |  |  |  |  |  |  |  |  |
|  | Bronchopulmonary dysplasia^a^ |  | 1.65 (1.42–1.92) | <.0001 |  | 1.12 (0.97–1.29) | .12 |  | 1.33 (1.22–1.45) | <.0001 |  |
|  | Congenital heart disease^b^ |  |  |  |  |  |  |  |  |  |  |
|  | Hemodynamically significant (surgery) |  | 4.33 (3.75–5.00) | <.0001 |  | 2·98 (2·62–3·94) | <.0001 |  | 4·42 (4·10–4·76) | <.0001 |  |
|  | Hemodynamically non-significant |  | 2.52 (2.29–2.76) | <.0001 |  | 2·14 (1·99–2·32) | <.0001 |  | 2·16 (2·06–2·28) | <.0001 |  |
|  | Other underlying medical disorders^a^ |  |  |  |  |  |  |  |  |  |  |
|  | Pulmonary hypertension |  | 3.85 (2.73–5.43) | <.0001 |  | 3.26 (2.44–4.34) | <.0001 |  | 2.71 (2.18–3.38) | <.0001 |  |
|  | Congenital lung disease and/or bronchial abnormalities |  | 4.34 (3.17–5.92) | <.0001 |  | 2.61 (1.90–3.57) | <.0001 |  | 3.98 (3.33–4.76) | <.0001 |  |
|  | Congenital tracheoesophageal fistula |  | 3.78 (2.73–5.22) | <.0001 |  | 1.78 (1.27–2.50) | .002 |  | 3.67 (3.01–4.49) | <.0001 |  |
|  | Cystic fibrosis |  | 2.32 (1.56–3.44) | <.0001 |  | 1.80 (1.26–2.57) | .002 |  | 4.63 (3.83–5.59) | <.0001 |  |
|  | Cardiovascular disease during perinatal period without CHD identified in the follow-up |  | 1.49 (1.33–1.66) | <.0001 |  | 1.47 (1.34–1.60) | <.0001 |  | 1.31 (1.24–1.39) | <.0001 |  |
|  | Cardiomyopathy |  | 3.42 (2.07–5.63) | <.0001 |  | 2.78 (1.78–4.34) | <.0001 |  | 3.99 (3.02–5.27) | <.0001 |  |
|  | Diaphragmatic hernia |  | 3.30 (2.11–5.18) | <.0001 |  | 2.99 (2.01–4.44) | <.0001 |  | 2.96 (2.25–3.90) | <.0001 |  |
|  | Omphalocele |  | 1.01 (0.52–1.97) | .97 |  | 1.03 (0.58–1.84) | .91 |  | 1.24 (0.84–1.82) | .29 |  |
|  | Muscular dystrophy |  | 6.69 (4.32–10.36) | <.0001 |  | 4.11 (2.77–6.10) | <.0001 |  | 7.29 (5.65–9.40) | <.0001 |  |
|  | Congenital abnormalities of nervous system |  | 1.96 (1.41–2.72) | <.0001 |  | 1.26 (0.92–1.74) | .15 |  | 1.91 (1.60–2.29) | <.0001 |  |
|  | Cleft palate |  | 1.35 (1.02–1.78) | .038 |  | 1.45 (1.16–1.81) | .001 |  | 1.75 (1.53–2.00) | <.0001 |  |
|  | Down syndrome |  | 4.03 (3.30–4.91) | <.0001 |  | 3.10 (2.58–3.72) | <.0001 |  | 3.39 (3.01–3.82) | <.0001 |  |
|  | Other chromosomal abnormality |  | 2.78 (2.14–3.61) | <.0001 |  | 1.80 (1.40–2.32) | <.0001 |  | 2.78 (2.38–3.24) | <.0001 |  |
|  | HIV infection |  | 3.61 (1.71–7.62) | <.001 |  | 2.17 (1.04–4.53) | .039 |  | 2.53 (1.64–3.92) | <.0001 |  |
|  | Solid organ transplant |  | 16.28 (5.12–51.75) | <.0001 |  | 5.85 (1.78–19.25) | .004 |  | 14.61 (7.28–29.33) | <.0001 |  |
|  | Stem cell transplant |  | 4.82 (1.39–16.70) | .013 |  | 6.22 (2.71–14.26) | <.0001 |  | 7.01 (3.97–12.36) | <.0001 |  |
|  | Maternal disorders during pregnancy: |  |  |  |  |  |  |  |  |  |  |
|  | Maternal smoking |  | 1.41 (1.32–1.52) | <.0001 |  | 1.40 (1.32–1.47) | <.0001 |  | 1.36 (1.31–1.41) | <.0001 |  |
|  | Cardiovascular diseases |  | 1.41 (0.99–2.00) | .055 |  | 1.42 (1.08–1.88) | .013 |  | 1.27 (1.05–1.53) | .015 |  |
|  | Diabetes mellitus |  | 1.22 (1.14–1.31) | <.0001 |  | 1.06 (1.00–1.12) | .046 |  | 1.10 (1.06–1.14) | <.0001 |  |
|  | Results are expressed as odds ratios (95% confidence intervals).  Abbreviations: CHD, congenital heart disease; GA, gestational age; ICD–10, 10^th^ revised edition of the *International Classification of Diseases*; LRTI, lower respiratory tract infection; NICU, neonatal intensive care unit; RSV, respiratory syncytial virus.  ^a^ICD–10 codes identified by hospital records.  ^b^CHD was identified according to the criteria from the Anatomic and Clinical Classification of Congenital Heart Disease and corresponding to validated ICD–10 codes. Cardiac surgery was identified as hemodynamically significant CHD.  Multivariate logistic regression models were developed using Generalized Estimating Equations (GEE) with exchangeable correlation structure. Odds ratios were adjusted for all presented covariates as well as region of maternity wards and environmental risk factors. | | | | | | | | | |  |
